# Digital Health Testbeds in Sweden: An exploratory study

**DOI:** 10.1177/20552076221075194

**Published:** 2022-02-14

**Authors:** Md Shafiqur Rahman Jabin, Evalill Nilsson, Anna-Lena Nilsson, Patrick Bergman, Päivi Jokela

**Affiliations:** 1 Department of Medicine and Optometry, eHealth Institute, 4180 Linnaeus University, Kalmar, Sweden; 2 Faculty of Technology, Department of Informatics, 4180 Linnaeus University, Kalmar, Sweden

**Keywords:** Testbeds, e-health, Complexity theory, Complex Adaptive System, Agents, Diversity, Connections, Adaptation, Perturbation, Path dependence

## Abstract

**Objective:**

This study explored the Swedish digital health testbeds through the lens of complexity science.

**Methods:**

The purposive sampling was used to identify 38 digital health testbed organizations to conduct interviews in written or audio-conferencing. The interview responses were aggregated and analyzed using thematic analysis. The themes were mainly generated through complexity theory and the principles of complex adaptive systems.

**Results:**

Fifteen testbed organizations responded, comprising 13 written responses and two audio-conferencing. Five main theoretical themes were generated: agents and diversity, connections and communication, adaptation and learning, perturbations, and path dependence. Agents and diversity depicted different types of testbeds, stakeholders and innovation, and the primary function and purpose of the testbeds. Various factors enhancing connections and communications among multiple stakeholders were identified, such as the quality of e-health solutions and the 2030 Agenda for Sustainable Development. Some adaptation and learning factors, such as internal reorganization, sharing and creating learning opportunities, and additional funding, guaranteed the sustainability of testbeds. Perturbations were characterized by two factors: non-linear interactions – lack of commitment and transparency in stakeholders' engagement, and uncertainty about testbed definitions and concepts. Path dependence highlighted the importance of history, such as previous positive and negative experiences.

**Conclusion:**

This study provided insights into testbeds' organization, their functions, how various aspects were challenged, and how they adapted to overcome and improve the system issues. Identifying the stakeholders and relevant factors, commissioning an evaluation, backing up with a contingency plan, securing adequate funding, and disseminating the findings can improve the testbeds' design and implementation.

## Background

Modern healthcare systems comprise a complex socio-technical system in which human behavior, performance, and culture are intimately associated with digital systems, aiming solely to improve healthcare quality and safety.^1^ The simultaneous increase in the demand for digital transformation in other industries, such as travel, banking, and manufacture,^2^ has transformed this socio-technical system, making it more digitally dependent on escalating cost, administrative, and logistical perspectives, and rising public expectations.^2,3^ This concept finds support in a previous study by Jabin et al. “Following only slightly behind, there has been an exponential increase in the possibilities offered by, and in the use of, digital technologies, both in individual devices and at a systems level”.^4^ However, implementing a new system in a healthcare organization is always complex, mainly from administrative and logistical perspectives. A number of issues arise, since end-users, including healthcare professionals within and outside of healthcare facilities, have some degree of ownership. This degree of ownership may appear as resistance and obstruction to the deployment of an innovation.^3^ A Swedish study found that, while government agencies were very optimistic about implementing new technologies in healthcare, the unstructured implementation process (without proper planning) and incoherent evaluation model (without compatible designing) indicated inequality of access to such new technologies.^5^ Similar findings suggested that the implementation of such technologies can even hinder and jeopardize health equity^6^ and patient safety,^3,4,7,8^ up to and including casualties.^9^ Several factors, such as international context, cultural differences, workflow inquiry, and adaptation to local situations, must be addressed in the context of implementation science. These factors may markedly affect the outcomes of the implementation process of digital and welfare technology.^10^ Consequently, building innovative and sustainable digital solutions for healthcare requires rigorous testing and evaluation of products and services in a real-world environment. Both healthcare professionals and patients can participate in an iterative design process.

In this context, digital testbeds are critical components, as they offer an opportunity for a range of stakeholders, including academics, industry experts, researchers, problem owners, and users, to gather, facilitate and develop innovation, research, and business through local, national, and international collaboration and cooperation.^11^ Collaboration is also the core concept of many Swedish testbeds. This objective is realized in a strategic project, the “Swedish Testbeds”, designed for stimulating dialogue, coordination, and collaboration nationally and internationally in order to disseminate and commercialize innovations within the field of digital testbeds.^11^ This innovation has been designed under the auspices of a government agency, Vinnova, responsible for administering state funding for research and development. A typical Swedish digital health testbed project is characterized by the involvement of different stakeholders, such as academia, industry, government, and the healthcare environment.^12^ The primary aim is to improve person-centered care, social care, and elderly care in different settings, including hospital, home care, and academic settings.^11^ The structure or model for the cooperation of the stakeholders is often followed by a triple helix construct,^11^ or a quadruple helix model of innovation.^12^ In the model that was suggested by Hasche et al. the quadrupole helix is viewed as a network of relationships, where combined resources and joined activities create value for the involved public and private actors.^12^

Several actors and phases may be involved in designing a framework for such testbeds or a testbed program: a dedicated core team, teams for collaboration, the testing phase, evaluation, information governance,^13^ interoperability,^14^ cybersecurity,^15^ communications, and commercialization.^2^ For the convenience of the reader, the term “testbed” will mainly be used, instead of “testbed organization”, throughout the article.

Even if the demand for testbeds is widely acknowledged, the definitions and concepts regarding testbeds are still diverse. Even the spelling of testbed varies across various literature, for example, “test bed”^16^ or “testbed”, first defined in a 1914 edition of *Webster's New International Dictionary*.^17^ According to Merriam-Webster's current definition, a testbed (spelled “test bed”) is “a vehicle (such as an airplane) used for testing new equipment (such as engines or weapons systems). Broadly, it is defined as “any device, facility, or means for testing something in development”.^16^ Sweden's innovation agency Vinnova provides information about the hundreds of testbeds available in Sweden — academia, industry, and the public sector, such as healthcare. Vinnova defines testbed as “a physical or virtual environment in which companies, academia, and other organizations can collaborate in the development, testing and introduction of new products, services, processes or organizational solutions in selected areas”.^11^

How the concept of the testbed is understood depends on the type of testbed, the discipline under which the testbed is designed or implemented, and the testbed's purposes and functions.^18^ In Sweden, testbeds are generally categorized as real, simulated, or laboratory-based testbeds; the word “lab” is often used to denote laboratory-based testbeds. A 'real testbed' is a real user environment where the technology is tested in and by the activity or organization to use such technology. A 'simulated testbed' is a simulated user environment, i.e., the test environment simulates real-life conditions for testing or demonstrating products, services, or processes at a system level. A 'laboratory-based testbed' is a controlled environment where individual functions or abilities are tested.^11^

In this exploratory study, we aim to gain insight into the concepts and use of testbeds within the context of digital health, and generate information for designing a framework within healthcare and welfare under the auspices of the “eHealth Arena”. The term e-health is defined as the healthcare services supported by electronic processes and communication, and is often used interchangeably with another term, 'digital health'.^19^ The “eHealth Arena” is a large-scale project, a triple-helix construct/model for cooperation among academia, the healthcare sector, and regional government to support, coordinate, and evaluate the use of e-health services and products in Kalmar County, Sweden. This project can merge business growth and new digital technologies and models of care to strengthen research, technological development, and innovation in real-world healthcare settings. Implementation of a testbed with such a scope is being planned for broad patient pathways, focusing on disease prevention, transfer of care to the home, and data sharing, accompanied by the vision of a long-term self-management practical perspective.

So far, very little research has been conducted regarding testbeds in Sweden used in the context of digital health. Therefore, there is a need for qualitative analysis to explore the various aspects and perspectives involved, as well as to inform the design and implementation of future testbeds in the same context. Moreover, a conceptual framework of complexity theory and complex adaptive systems (CAS)^20,21^ is suitable to analyze and interpret digital health testbeds.

The overall aim of this study is to explore digital health testbeds in Sweden: their design, implementation, and management, as well as the main drivers of and challenges for testbed development. This study investigates the following research questions:

1. What are the characteristics of digital health testbeds in Sweden, and how do testbed stakeholders define the primary function and purpose of the testbeds?
2. What were the main benefits facilitated by the testbed projects, as perceived by the testbed stakeholders?
3. What were the main challenges, as perceived by the testbed stakeholders?
4. What were the outcomes of the testbed projects?

## Theoretical framework

Complexity science deals with system thinking and studies complex systems.^20^ The tools and models of complexity science provide insight for understanding and observing a system, mainly where system transitions occur.^21^ Over the last three decades, among all other disciplines, healthcare has become one of the most complex socio-technical systems, experiencing changes continuously. With the advancement of technological and clinical innovation, the influx of patients to hospitals has increased, as has the multidisciplinary approach of healthcare services.^22^

Complexity science propounds that healthcare organizations are complex adaptive systems with a set of connected or independent agents, including patients and care providers acting knowingly to attain a common purpose.^23^ This also illustrates the science of non-linearity and interactions, where unforeseen and uncertain outcomes are recognized in routine practices. Healthcare organizations themselves are constantly changing the situation in the workplace and the entire organization.^24^ This field of science has been applied in healthcare to improve care coordination for complex pediatric patients^25^ and to safeguard patients from implicating team training in healthcare.^26^ Moreover, complexity theory has been used in health services research as a theoretical framework to better understand complex situations, such as complexity account for rates in staff turnover,^27^ and to conceptualize work environments^28^ and primary care organization.^29^

As we study settings and infrastructures that involve multiple stakeholders, a systemic view can help us understand the interconnected human activity systems and technologies applied to make the whole testbed system work. The interactions between the system components and collaboration within the complex system focus on a specific branch of systems thinking when CAS is studied, and complexity theory can be applied.^30,31^ By recognizing different perspectives, we introduce a crucial aspect of complexity into situations, with the eventual aim of the investigation, consensus, and improvement. The inter- and multidisciplinary testbed stakeholders have been studied through the lens of complexity science, using attributes of complexity theory and features of CAS as characteristics. It is not enough to understand the underlying dynamics and emergent behavior of the system alone. This should be accompanied by an understanding of the system at various levels, including localized interactions of the individuals within teams, or networks within larger organizations.^32^

In this study, the testbeds and their stakeholders are characterized and analyzed according to the attributes that are described in Table 1: agents and diversity, connections and communications, adaptation and learning, non-linear interactions and unpredictability, and path dependence.^31,33^

**Table 1.** The selected nattributes to characterize digital health testbeds in Sweden.

Attributes	Description
Agents and diversity	A complex system is heterogeneous; it comprises many different parts, i.e., agents. The agents act autonomously, but they are also interrelated and interdependent; the actions of one agent affect the others.
Connections and communication	A complex system is open, and it interacts with its environment. The agents within the system communicate with each other and also with the environment.
Adaptation and learning	An open system is capable of reacting and responding to external changes in its environment. Internal adaptivity within the system facilitates learning and cooperation between the agents.
Perturbations: non-linear interactions and unpredictability	Small internal and external changes can lead to large and unpredictable outcomes in the system.
Path dependence	The system is sensitive to initial conditions.

## Methodology

This is an exploratory qualitative study. The study participants were selected by using purposive sampling, where the principal investigator made an informed decision of the selection. The data was collected using four open-ended interview questions, and the responses were analyzed through thematic analysis with deductive and semantic approaches.^34^

### Sampling: selection of testbeds

The principal investigator made a list of digital health testbeds in Sweden in both academic and industry settings. Firstly, each Swedish university and college was listed, and their engagement with any testbeds was checked by visiting their website. Other approaches of listing testbeds involved information from Vinnova, combined with testbeds found via Google searches as well as a 2018 report by consulting firm Kontigo, entitled “*Slututvärdering av Projekt Innovationssluss 2.0*”.

A total of 38 digital health testbed organizations were finally selected. Of these, several were not single testbeds, but rather a collaboration of multiple testbeds. Each testbed website was visited to create a list of testbed owners, researchers, and relevant contact persons. Contacts and email addresses for all 38 testbeds were obtained from the relevant testbed websites. Two testbed contacts could only be contacted through their websites rather than email addresses. Follow-up communications were conducted via emails as necessary, for example, if any additional information on the type of testbeds were needed from the participants.

### Methods of data collection

This phase involved developing a set of interview questions based on the overall aim of this study and the specific research questions. The primary purpose was to explore how digital health testbeds in Sweden are designed, implemented, and managed. To conduct the interviews, a standard email protocol was developed in English, including the following four open-ended questions:

1. What definition of testbeds did you use within your work? Which stakeholders were included in your testbeds?
2. What methods/methodology did you use to develop/design your testbed (for example, action research methods or other methods)? Why did you choose such methods?
3. What were the challenges you faced during or after your testbed's design, implementation, and management?
4. How did/does your testbed benefit the population or achieve its purpose? Did you reach your goals?

The email also explained the study's aim and scope, and provided relevant information, including website links and participant information sheets with the attached informed consent form.

The participants could reply to the questions in writing and send answers by email. Alternatively, they could choose a semi-structured interview via phone or audio conversation, for example, Zoom (without video), a teleconferencing software program. Written interview responses in Swedish were translated into English by a research assistant with English proficiency and cross-checked by the co-authors. The semi-structured interviews were transcribed by the principal investigator. Both types of interview responses were collected, aggregated, and recorded by the principal investigator in Microsoft Spreadsheets for analysis.

### Methods of analysis

The interview responses were analyzed by the principal investigator using thematic analysis. The approach was mainly deductive, as the collected interview data were analyzed, and the themes were generated through the theoretical lens of complex systems, as presented in Table 1. Moreover, the coding and theme generating were conducted on a semantic level, i.e., based on the interviews’ explicit content. No assumptions were made regarding the latent underpinnings of the data.^8,35^

The thematic analysis comprised familiarization with the narratives by iterative reading and examining data, and coding by labeling words, phrases, or sentences to extract potentially relevant concepts. The list of concepts was then reviewed to conceptualize broader patterns, i.e., themes created by bringing a few or several concepts together, and labeled based on the processes, differences, and potential connections between them. A list of themes of similar nature was further grouped and related to the parent attributes in the theoretical framework.^8,35^

The deductive analysis consisted of three sub-analyses. Firstly, data were analyzed under the common norm of type or category of testbed organizations. For example, if the designated (interviewed) testbed was a single testbed, or a combination (or a collaboration) of multiple numbers of testbeds under the auspices of the same title. The testbeds were also classified by type (real, simulated, and laboratory), or as a combination of any two or all three types of the testbed. Moreover, the definition used to characterize the testbeds, and the model of innovation used to design and implement the testbeds, were also described (see Table 2).

**Table 2.** Type of participants’ response and statistics of response to each interview question.

Testbed	Type of response written/ telephone	Type of response English/ Swedish	Type of response complete/ partial	Response to the questions			
				Q1	Q2	Q3	Q4
TB₁	Written response	English	Partial	✓	**O**	✓	✓
TB₂	Written response	English	Complete	✓	✓	✓	✓
TB₃	Written response	English	Complete	✓	✓	✓	✓
TB₄	Written response	English	Complete	✓	✓	✓	✓
TB₅	Written response	English	Complete	✓	✓	✓	✓
TB₆	Written response	English	Partial	✓	X	X	X
TB₇	Written response	English	Partial	✓	✓	✓	**O**
TB₈	Written response	Swedish	Complete	✓	✓	✓	✓
TB₉	Written response	Swedish	Partial	**O**	✓	✓	**O**
TB₁₀	Written response	Swedish	Complete	✓	✓	✓	✓
TB₁₁	Written response	Swedish	Complete	✓	✓	✓	✓
TB₁₂	Written response	Swedish	Complete	✓	✓	✓	✓
TB₁₃	Written response	Swedish	Complete	✓	✓	✓	✓
TB₁₄	Telephone interview	English	Complete	✓	✓	✓	✓
TB₁₅	Telephone interview	English	Complete	✓	✓	✓	✓

✓ = answered completely; X = did not answer; **O = **answered partially

Secondly, complexity theory as a conceptual framework was applied to study the testbed organizations’ phenomena and classify them under the parent attributes, as shown in Table 1.^36^ Finally, principles of CAS^20,30^ were applied to investigate the team characteristics and behavior of the actors involved, including testbed owners, managers, employees, stakeholders, and other professionals interested in the design and implementation of the testbeds.

## Results

Fifteen responses, made by one participant per testbed organization, were received out of the total 38 selected. Of these fifteen responses, eleven were considered complete responses, and four were partial. A response was deemed partial if the participant did not respond to all four questions, or only partially responded to any one of the questions. All responses, including partial responses, were considered for analysis. Of all responses, 13 responses were written, and two more were conducted through audio conversation. Of the 13 written responses, seven were in English and six in Swedish, which were translated into English. Moreover, seven follow-up communications were conducted in English via email to clarify some points in the written responses. Detailed information about the type of responses and statistics of responses to the interview questions is provided in Table 2.

The free-text narratives of the 15 interviews were analyzed, of which 474 phrases or keywords (see Appendix A) were extracted, which described 194 potentially relevant concepts. These concepts were grouped into 49 themes, which were finally aggregated and related to the parent attributes in the theoretical framework (see Appendix B). The findings are presented under the rubric of parent attributes of complexity theory and CAS principles, and elucidated in more detail by using the more fine-grained themes and concepts that were generated from the empirical data. In addition, a summary of the benefits, challenges, and outcomes of the testbed projects is presented.

### Agents and diversity

The theoretical term 'agents’ refers to the stakeholders of a testbed, both organizations and individuals, and it can also include the technologies that are used within the testbed. Moreover, agents can also mean different testbeds that are clustered in multiple testbeds. In a complex system, diversity implies that the testbeds themselves be of different types, and comprise different agents; the various agents can define and perceive the testbeds differently. The agents can act autonomously, but simultaneously; they are interrelated and interdependent, so that the actions of one agent affect the others.

The following section describes how each testbed was organized – the type of testbed, type of stakeholders involved, type of definition used, and primary purpose. Table 3 illustrates some key characteristics of the fifteen testbeds.

**Table 3.** Key characteristics of the testbeds.

**Testbed**	Type of testbeds *Single/Multiple* Real/Lab/Simulated	Type of stakeholders Innovation model (T/Q)	Primary function	Purpose of the testbeds
TB₁	*Single* Real/Lab/Simulated	A business organization and a research institute (-)	-	- Improve the quality of care delivery or service - Develop, test, or create demand for e-health solution, product, or service
TB₂	*Multiple* Simulated	Academic institutions, research institutes, public & business sectors (T)	- A collaborative meeting place - A technological testing place	- Improve the quality of care delivery or service - Develop, test, or create demand for e-health solution, product, or service - Build a collaborative environment
TB₃	*Multiple* Simulated	Research institute, health (elderly) care, and government organizations in three municipalities (T)	-	- Improve the quality of care delivery or service - Develop and support business growth
TB₄	*Multiple* Real/Lab/Simulated	Academic institutions, research institutes, business, government, and public organizations (Q)	Formal definition	- Improve the quality of care delivery or service - Develop, test, or create demand for e-health solution, product, or service - Develop research and education
TB₅	*Single* Real/Lab/Simulated	Academic institution and company (-)	-	- Build a collaborative environment - Develop research and education
TB₆	*Multiple* Real/Lab/Simulated	Four regions’ academic institutions, research institutes, healthcare, business, and government organizations; and industry, technical and market analysts (Q)	- Testbed as a context - A collaborative meeting place - A technological testing place	- Improve the quality of care delivery or service - Build a collaborative environment - Develop and support business growth
TB₇	*Multiple* Simulated	Three regions' academic institutions, government organizations, and research institutes; and healthcare system developers (Q)	- Testbed as a context - A technological testing place	- Improve the quality of care delivery or service - Develop, test, or create demand for e-health solution, product, or service
TB₈	*Single* Real/Lab/Simulated	An academic institution, government, business, and public organizations; and Healthcare professionals and innovators (Q)	- A collaborative meeting place	- Improve the quality of care delivery or service - Build a collaborative environment
TB₉	*Single* Real/Simulated	Health (elderly) care and business organizations, and academic institution; and researchers (-)	- Formal definition	- Improve the quality of care delivery or service - Develop, test, or create demand for e-health solution, product, or service - Develop and support business growth
TB₁₀	*Single* Real	An academic institution, research institute, government, and public organizations (Q)	- Formal definition	- Improve the quality of care delivery or service - Develop, test, or create demand for e-health solution, product, or service
TB₁₁	*Single* Simulated	An academic institution, Healthcare, public, and business organization (T)	- Testbed as a context - A technological testing place	- Improve the quality of care delivery or service - Develop, test, or create demand for e-health solution, product, or service - Develop and support business growth
TB₁₂	*Multiple* Real	An academic institution, Public, government, and business organizations, and research institute; and entrepreneurs (Q)	-	- Improve the quality of care delivery or service - Develop, test, or create demand for e-health solution, product, or service - Develop and support business growth
TB₁₃	*Single* Real/Lab/Simulated	Research institutes, business and government organizations; and a reference group (T)	- A collaborative meeting place	- Improve the quality of care delivery or service - Build a collaborative environment
TB₁₄	*Multiple* Real/Lab	Several Regions' academic institutions, research institutes, healthcare, government, and business organizations; and service designers, and informal research groups (Q)	- Testbed as a context - A technological testing place	- Improve the quality of care delivery or service - Develop, test, or create demand for e-health solution, product, or service - Build a collaborative environment
TB₁₅	*Single* Lab	Academic institutions and research institutes; and scientists and psychologists (-)	- Testbed as a context - A technological testing place	- Build a collaborative environment - Develop research and education

TB = Testbed; T = Triple helix; Q = Quadruple helix; (-) = Unknown

#### Types of testbeds

The testbed organizations consisted of either single or multiple testbeds. Of the fifteen responses that comprise the data set, eight were from single testbeds, and seven were from multiple testbeds. Multiple testbeds were a combination or collaboration of more than one testbed (see Table 3). For example, TB_4_ comprises a collaboration of 50–60 testbeds in western Sweden, and TB_6_ consists of three testbeds in three different municipalities.

Seven testbeds were in one specific category, of which three were real, three were simulated, and one was a laboratory. Eight testbeds were a combination of either two or three types, of which six were a combination of all three types, real, simulated, and laboratory, two were a combination of two types, real and simulated, and real and laboratory.

#### Types of stakeholders involved

In Table 3, the stakeholders are shown at an organizational level. Academic institutions included universities involved as stakeholders in 13 testbeds, whereas research institutes consisted of the research industry, science park, or science cities, and were identified in 11 testbeds. Business organizations comprised business sectors and hubs, and companies were apparent in 11 testbeds. The government organizations included government sectors and agencies, county councils, and municipality units, and were identified in seven testbeds. Healthcare organizations consisted of health and elderly care centers, and clinics, and were involved in six testbeds. The public organizations comprised public sectors, civil, and pensioner's organizations, and were apparent in five testbeds.

The typical individual stakeholders were healthcare professionals, healthcare system developers, service designers, innovators and entrepreneurs, market and technical analysts, and researchers. The stakeholders could also be smaller groups of individuals, such as reference groups or informal research groups.

Individual stakeholders were mainly engaged in testbed projects where they were needed to provide expertise. For example, a market analyst was hired to perform a market survey, and a psychologist to analyze narrative texts for research purposes.

The model of innovation adopted by these testbeds was classified into two categories (see Table 3): the triple helix model implies a collaboration among academia, industry, and government, and the quadruple helix model, an interaction between academia, industry, government, and civil society/ environment. Of 15 testbeds, seven adopted quadruple helix models, four adopted the triple helix model of innovation, and the remainder did not provide enough information to be classified.

#### Primary function and purpose of the testbeds

The above-mentioned definition of a testbed by Vinnova^14^ was adopted verbatim by three of the studied 15 testbeds to describe the primary function of testbeds. Generally, the agents perceived and defined their testbed's primary function in three different ways: as a context, a collaborative meeting place, or as a technological testing place for technology. The context refers to a facilitating infrastructure or environment; a collaborative meeting place emphasizes the agents’ collective effort to create healthcare solutions; while the testing place is more focused on technological innovation and iterative design process. In two cases, the testbeds did not use any specific definition. It is also important to point out that the function of the same testbed can be interpreted differently by different agents. The diversity of the definitions of primary function are shown in Table 3.

Five main purposes were identified. The primary purpose for most of the testbeds was to improve the quality of care delivery or service, across several dimensions, including safety, efficiency, effectiveness, patient-centeredness, availability, and equity. Other everyday purposes of testbeds included developing, testing, or creating demand for new and sustainable e-health solutions or services, and creating business growth for small and medium-sized companies. The fourth purpose was to build a sustainable collaboration environment or consortium that could support innovation and research within the digital health domain. The fifth aim was mainly focused on developing knowledge and opportunities through research and education.

### Connections and communication

A complex system implies a variety of internal connections and interactions between agents, as well as an openness and ability to interact with that system's environment. An open system also emphasizes the dynamic characteristics of testbeds. As an open system does not have an equilibrium state, the connections are flexible and versatile, and new connections can continuously emerge from internal and external communication and interactions.

In the studied testbeds, various connections and communications were identified, both on an organizational and personal level. Organizational-level connections involved academic, public, business, healthcare, pensioners, and county councils. Personal-level connections involved partners and colleagues through previous associations. A range of communications, and their impact, were reported, for example, information exchanged by two parties signing an agreement. These communications involved information flow, information exchange, conversation, and interactions. Connections between stakeholders, partners, or collaborators were expanded later in some cases, while others were reduced according to the rise of new needs or an adjustment of the project's scope.

Two primary principles for engaging in and improving further collaboration for various team members were identified: the concept of testbeds and the management of testbeds. A few concepts, such as 'plug-and-play' playground, and 're-creation' of testbeds instead of institutionalizing them, led to open collaboration. Moreover, the concept of value co-creation, i.e., the needs of the users and owners, and matching organizational requirements with entrepreneurial ideas, strengthened product development, innovation and service, and project partnership. Creating demand or value further encouraged team members to integrate and share e-health applications within the organization.

The testbeds’ governance or management was another essential principle that drove team members to perform their daily business operations and test solutions. For example, one group of testbeds was managed by the existing line of organization in the respective municipality. For routine operations and several successful deliverables, the management in the appointed department further empowered the organization's colleagues to gain new experiences and tools for a future project.

Healthcare services and research are examples of external factors that could influence collaboration, impacting cooperation and building new partnerships. For example, healthcare services enabled active collaboration with other research institutes or industries, business organizations, pensioners’ organizations, and academic institutes. Healthcare was looked at through the lens of value creation from the user perspective. The healthcare system was required to integrate resources to treat those values as patient resources and the network around the patients, to create further resources. For example, doctors, nurses, and other healthcare professionals needed to build a network around patients and integrate resources.

The research for further partnership with other research institutes or academic institutes influenced collaboration. The association was later extended, and involved the respective municipalities’ research institutes, such as science parks, through private initiatives, since these institutes (in many cases) represent and host testbeds regionally.

Two important attractors that facilitated collaboration in the studied testbeds were the quality of e-health products, services, and solutions, and critical aspects of the 2030 Agenda of Sustainable Development. The desired quality improvement of the care delivery and e-health products and services, especially elderly care, was an important driver for commencing and further reshaping the collaborations. With the key aspects of Agenda 2030 in focus, the testbed operation was sped up for collaboration and commercialization, which further made the testbed last with sustainable value propositions and strengthened further cooperation.

### Adaptation and learning

The sustainability of complex systems is based on their adaptability, i.e., their ability to adapt and respond to internal and external challenges and changes. Internal adaptivity facilitates learning and cooperation among agents. An open system is capable of reacting to external changes in its environment.

In the studied testbeds, different types of adaptation to complexity for improvement were apparent: system adaptation, adaptation to innovation, adaptation of new strategies, and internal reorganization. System adaptation included adaptation to a norm of visual perception, 'plug-and-play' playground, entrepreneurs’ line of thought, and making use of employees’ experiences, virtual testbed environment, the real needs of the owners, and the interdisciplinary research environment. Adaptation to innovation included testing new methodologies for creating change as innovation initiatives and innovation hubs.

When the technology, cost of implementation, and distance between stakeholders prevented them from finding solutions, new collaboration strategies and methods were introduced to overcome these drawbacks. Some examples included mounting a camera to a physical testbed for remote viewing; involving only the testbed players/ stakeholders who should be concerned; creating virtual IT testbeds environment for communication; participating in overseas projects; and testbed initiatives. This, in turn, improved operations, collaboration, and communication.

In many situations, the testbed stakeholders were guided by internal reorganization. For example, two focus areas, namely information technology and mobility, were attained through internal reorganization. This helped the stakeholders understand which activities they could operate individually, and which to work together on shared or similar interests, i.e., where collaboration should be the critical factor. The stakeholders’ expertise was shared or acted upon to restore contacts, project and technology management, and communication.

Testbed professionals learned from each other, and shared the lessons learned with each other through interaction and collaboration. The testbeds facilitated various activities, for example, the primary area included connections, communications, and collaboration activities among the testbed agents and the testbed environment. Another area comprised the testing and evaluation of e-health services. Moreover, research and education-oriented activities were enabled by testbeds. Finally, the activities were related to the testbeds’ management.

During interaction and collaboration, the testbed professionals also experienced several challenges. The challenges could be related to policy and guidelines, such as what rules to apply, issues of payment model, agreement documents, and legal aspects. On the organizational level, some challenges emanated from the lack of commitment from organizations and their top management, and the lack of trust between the stakeholders. On the operative level, challenges were often time and resource constraints, due to the busy healthcare environment, for instance, the lack of time for the healthcare professionals to dedicate outside their regular working hours. Heavy workload also entailed difficulties engaging and retaining skillful staff and experts, and caused frustration among healthcare employees during the testing and implementation of digital solutions.

There were many uncertainties concerning the testbeds’ goals, focus, and standard rules. Moreover, environment-related challenges implied a lack of access to healthcare sites for research, which further hampered the development of sustainable healthcare products. Challenges could also be based on a lack of communication, which was enhanced when the stakeholders were from different organizations. Also, insufficient matching between healthcare needs and entrepreneurial ideas could compromise the sustainability of the testbed environment. Finally, the technology itself could be perceived as an obstacle that prevented finding solutions. These issues included the high cost of implementation, low technology acceptance, and steep learning curve.

However, these challenges paved the way for creating learning opportunities and sharing insights, even among international collaborators. There was also evidence of workplace learning and the extension of collaboration through traveling, meeting new potential partners and stakeholders, and sharing research collaboration expertise at a broad level. Some of these issues were improved through system adaptation, such as using entrepreneurs’ line of thought, employers’ experience, real needs of the owners. Other challenges were overcome by adopting new strategies or methodologies, such as mounting a new camera so that the physical testbed could be seen from a distance and creating virtual IT testbed environments to test systems under development.

The study identified several ameliorating factors that were associated with the testbed projects. The most frequent factors were 'additional funding' and 'learning and insight'. The funding could be received from the government, private funding agencies, or innovation agencies. Learning and insight resulted from new learning opportunities that the testbed project offered and the shared lessons learned among the involved stakeholders. On the individual level, learning to use new digital tools could imply empowerment. In this context, successful communication and collaboration must be considered pre-conditions for mutual learning.

Other ameliorating actions included internal reorganization, adaptation to more sustainable business models, and generating legal guidance for implementing digital solutions on the organizational level. At later phases, the need to acquire extra research equipment, receive a permit to work at a hospital, retain skillful staff, increase productivity were achieved through various testbed initiatives, collaboration, and participation in other funded projects. Other activities included attaining mobility and focus areas, managing the application of information technology, and restoring contacts, projects, and communication. The desired outcome was to make the testbeds last, with sustainable value propositions and robust facilitation processes.

### Perturbations: Non-linear interactions and unpredictability

The diversity and versatility of a complex, open system are essential for system adaptability, but they also increase the system's uncertainty and interactions with the environment. In the same way, because an open system does not have an established equilibrium, and new connections can continuously emerge, the effects of the interactions are unpredictable, and they can be non-linear. Therefore, small internal or external changes can lead to large outcomes, or vice versa.

The studied testbeds were characterized by uncertainty, for example, uncertainty about the rules that should be applied, concepts of healthcare, project goals, and definition of the testbed, industrial focus, and business model.

Two features related to non-linear and unpredictable interactions were difficulties in stakeholders’ involvement, and openness in interactions. It wasn't easy to constantly regain commitment, or to reach out to the stakeholders internally, within the testbed organizations, and externally, within business and academia. There were also challenges in obtaining the different parties and (healthcare) stakeholders involved in product development, even after exerting a lot of time and effort. These were due to several factors, such as a lack of time, interest, and motivation of the healthcare professionals, as well as a lack of awareness of the testbed concept.

Another feature that contributed to unpredictable interactions was the lack of transparency both internally and externally. In general, establishing new operations for a testbed demands continuous communication, because the foundation must create trust with businesses and secure their involvement. A lack of openness among internal or external stakeholders deviated from the testbed's purpose, reducing likelihood of testbed implementation and creating mistrust among team members, deteriorating routine operations.

Difficulties in reaching out to both internal and external stakeholders, supplemented by the ambiguous and vague concept of testbeds, challenged the decision-makers regarding the testbeds’ overall benefit. Moreover, testbed managers sometimes ignored the limits of their own abilities to handle pressure, and involved themselves in various activities without balancing internal and external communications. This eventually made them exhausted and frustrated, resulting in rearranging procurement and managing the organization's entire process. The team members complemented each other through additional work to adjust the innovation process for improvement.

### Path dependence: The importance of history

The initial conditions of a complex system are essential, even if it may be difficult to predict their future consequences. Four aspects of the stakeholders’ and testbed project's history that impacted the ongoing collaboration were: previous negative experience, previous positive experience, association through previous partnership, and history of specific strategy or method.

Previous negative experiences incorporated some specific conditions. For example, losing control of testbed details by the researchers negatively impacted the later stages of the testbed development and implementation processes.

Previous positive experiences included how mutual consensus regarding using employees’ knowledge to set up testbed solutions and conduct market surveys improved the day-to-day operations and collaborations among partners. In addition, previous experience as a researcher in the former organization led to enhanced collaborations, and setting up an entirely new infrastructure for performing interdisciplinary experimentation and analysis.

A personal-level association further established a basic sense of trust and confidence through previous collaboration with a researcher from another organization. This enabled sharing of each other's expertise, and construction of a small infrastructure similar to that of an earlier organization. Previous positive experience and collaboration led the testbeds to achieve most of their project goals, or more than initially expected.

The most frequently used approaches were 'service design' and 'need analysis’. For instance, service design was successfully used as a model and methodological approach previously in welfare technology, which encouraged some stakeholders in the same municipality to adopt the same strategy. This service-logic perspective could create users’ needs, and create value around those needs, balancing internal and external resources in a way that proved to be a great success.

Furthermore, an essential foundation was established by the previously used methods of 'clinical trials’, strengthening and building up both interior ideas from the health care sector, and external requests for support and assistance to flourish, ultimately leading to the achievement of the project goals.

The guiding principles for the testbed projects, and the selection of specific models and methods, could be based on previous experience. Still, they may also originate from users’ and project owners’ needs, the project or business goals, or a usability evaluation of the project.

### Benefits, challenges, and outcomes of the testbed projects

 Table 4 illustrates the impacts of the testbeds, depicting the projects’ goals, facilitation, challenges, and ameliorations and outcomes achieved. The difference between the purpose of the testbeds, in Table 1, and the project's goal, in Table 4, should be noted with caution. “Purpose,” for example, provides a general idea of the testbed's aims and objectives, whereas the goal is the more focused target, specific to each testbed.

**Table 4.** Properties of the testbeds depicting project's goals, facilitation, challenges, and amelioration and outcomes.

Testbed (TB)	Project's goal	Facilitation	Challenge	Amelioration and *Outcomes
TB₁	-Developing clinical application for a 3D visualization solution -Improving diagnosis, efficiency, patient safety, and quality of care	-Facilitating research on medical images/visualizations -Performing the technical implementation (by the company)	-No controls over testbed details by the researchers at the early stage	-Taking complete control of the testbed details by the researcher at the later stage *Several scientific publications
TB₂	-Creating, testing, and developing solutions through the internet of things (IoT) and collaboration -Increasing service availability, sustainability, and safety in the community	-Facilitating tests in an actual collaborative environment by sharing the latest technology without owning any infrastructure -Helping partners with communication and technology management	-Low testing of products since the inauguration -Uncertainty about what rules needed to apply by the stakeholders and company	-Internal reorganization -Revising the team plan -Sharing lessons learned *Became a collaboration arena
TB₃	-Testing pre-market digital solutions and improving their efficiency and effectiveness used in elderly care -Creating growth for small and medium-sized companies	-Facilitating a meeting place for innovators, entrepreneurs, and healthcare professionals -Providing digital solutions based on clients' needs by pre-testing solutions -Governing existing line of organization in the respective municipalities	-Commitment from top management -Maintaining a steady flow of entrepreneurial ideas and solutions to match with organizational needs -Time- and resource-consuming process	-Creating a lot of learning opportunities *Contributing to lots of learning opportunities
TB₄	-Developing sustainable solutions for societal challenges -Building knowledge and driving healthcare quality through research and education	-Extending collaboration to a new level in various regions	-Sustainability of the project (two years) -Industry focus	-Speeding up commercialization -Making the testbeds last over time with sustainable value propositions *The project is still running
TB₅	-Creating a research environment with a focus on IoT -Collaborating with companies within the city and the region	-Facilitating management of daily operation by the appointed department -Developing innovative initiatives	-Communication, trust, and involvement of the stakeholders -Reduced number of clients (buyers) -People's time	-Empowering colleagues to gain experience for future projects *Several successful deliverables
TB₆	-Strengthening several regions' small- and medium-sized companies -Creating sustainable collaboration environments -Improving the quality of health and elderly care	-Facilitating the testing of new innovative solutions in health and elderly care -Conducting demonstration opportunities for innovative 5G products and services	-	- *The project is still running
TB₇	-Developing integration towards a health information architecture standard -Delivering services and creating rapid set-up of test environments -Improving the quality of deliveries	-Facilitating integration of medical records and medicines in the National Service platform -Developing an early error detection through interoperability testing -Assisting with pre-testing the solutions	-Technology itself that prevented finding solutions -Excessive cost of implementation -Distance between the stakeholders -Frustration of the employees -Long test description	-Providing stand-alone test scripts to run on their site for better implementation *Completing several assignments
TB₈	-Contributing to the wellbeing of the elderly and people with disabilities -Contributing to regional innovation -Developing innovator-friendly climate	-Involving of all stakeholders in the design of the testbed -Facilitating innovators to connect with users for developing the innovations -Assisting with evaluation and followed up on the business experience	-Legal guidance -Collection for agreement documents -Developing a payment model	-Receiving financial support from politicians -Appointing an evaluation researcher for legal guidance *Achieving many tested innovations
TB₉	-Managing sustainable innovations strategically and effectively -Testing and developing new digital health solutions -Developing competitive business in the region	-Facilitating the evaluation of usability and testing systems under development -Conducting external monitoring, including needs of clinicians, researchers, and business	-Reaching out to many external partners -Organization name recognition, both internally and externally -Concept of the testbed itself -Wasting excessive time and effort on the concept	-Receiving further funding from a government-based innovation agency *Becoming a regular line organization
TB₁₀	-Developing technical innovations and improving quality of care for elderly and disabled people -Increasing adoption of new technology with an understanding of new solutions	-Facilitating evaluation (need assessment) -Helping with user-centered development	-Involvement of all stakeholders -Wasting excessive time and non-productive effort on the business model -Very low testing of products -Payment model -National guidelines (legal aspects) -Demographic challenges -Conducting routine practices in the healthcare department	-Learning lessons to meet users' needs -Receiving additional funding from a government-based innovation *Achieving most of the project goals
TB₁₁	-Creating value for patients and employees through innovation -Developing and testing new products, services, processes, and solutions -Supporting private business by offering healthcare services	-Performing a pilot study, evaluation, and analysis for the testbed design -Facilitating research/ clinical trials -Helping the companies and people navigate the test opportunities -Forming transnational collaborations	-Vague goals (initially) -Sustainability of the testbed environment -Involving healthcare operation and staff in product development -Access to healthcare (professionals) -Retaining skillful staff and experts -Measuring the impact on the citizens	-Appointing a testbed coordinator for international collaborations -Receiving partial (extra) funding from the region -Participating in other projects *Achieving most of the project goals
TB₁₂	-Developing digital solutions by increasing business growth, individuals' capability, and quality of life -Ensuring equal health and efficiency in public health services	-Facilitating evaluation and testing of digital solutions -Developing knowledge and communication through research and education -Ensuring data access for the researchers	-Developing relevant documents -Legal aspects -Maintenance for the privacy of healthcare during the study -Obtaining access to healthcare (initially)	-Receiving permit to work at the hospital by the research team -Gaining lessons and insights *The research study is still not complete
TB₁₃	-Creating high demand for the development of new solutions and products -Building a consortium through a partnership with other organizations	-Offering courses, training, counseling, marketing, and networking -Facilitating evaluations to develop new products and innovations -Conducting several seminars	-Wasting excessive time and effort on the definition of testbed and business model	-Reaching a sustainable business model (after the project ended) *Developing a brochure for various solutions
TB₁₄	-Developing interregional collaboration for innovation -Creating value for healthcare services and improving their quality and safety -Meeting challenges and needs faced by healthcare services and bringing staff, patients, and families together for creating healthcare services	-Developing competence and value by understanding users' need -Facilitating management decisions in each organization for user-driven development -Sharing e-health applications and implementing research and education -Forming a network for doctoral students -Created both national and international collaborations	-Definition of testbed itself -The process of healthcare itself -Heavy workload and stress (at a later stage) -Shortage of staff -Uncertainty about healthcare's future -Distance between and movement among stakeholders	-Learning from the results -Receiving additional funding -Reorganizing to a more robust facilitation process *Accomplishing many projects and a big success with national and international impact
TB₁₅	-Creating opportunities and developing new research areas -Making research affordable and initiating new collaborations with other institutes	-Supporting with infrastructure, premises, and research equipment -Conducting demonstrations and workshops -Facilitating courses and training -Forming a network of doctoral students	-Research technical issues and inadequate research equipment	-Receiving an additional funding -Purchasing extra research equipment *Exceeding expectations and inspiring other institutions to have similar infrastructure

Empowering and enhancing collaboration among the various stakeholders was one of the main benefits of the testbed projects. Apart from collaboration, other important benefits the testbeds facilitated were testing and evaluating e-health products, solutions, and services. These facilitations comprised testing or pre-testing digital products and solutions, and assessing the need for digital services. Other benefits were the facilitation of research and education-related activities, such as courses, workshops, training, and competencies. The facilitation of management operation was also evident, such as governance and maintenance by an existing line of operations or an appointed department.

A number of external and internal challenges were encountered during the design and implementation of the testbeds. The challenges were incorporated with uncertainties about concepts, definitions, business models, focus, goals, rules and regulations, and the future of healthcare. These uncertainties were associated with time and resource constraints, such as excessive wasting of time and effort on the concept, or definitions of the testbeds. Also, some challenges complicated communication and collaboration among stakeholders, for example, lack of involvement and trust among stakeholders and the distance between them. Other challenges included policy and guideline related issues, such as legal aspects and payment model; technical challenges, for example, losing control over technical details; environment-related issues, for instance, lack of funding and lack of access to healthcare site for research; difficulties in e-health product development; and workforce-related issues, such as retaining skillful staff and experts.

Some of the challenges were tackled by applying for and receiving additional funds during or after implementing the testbeds. Other ameliorating factors included sharing lessons learned and creating learning opportunities; technical implementation and commercialization of e-health products or solutions; teamwork-related factors, such as empowering colleagues to acquire new knowledge and experience; and policy- and guideline-related solutions, such as appointing an evaluation researcher for legal guidance. Moreover, collaboration issues were overcome by participating in other funded projects and assigning a coordinator for international stakeholders. Organization and sustainability problems were addressed by reorganizing a more robust facilitation process to reach a sustainable business model.

Specific outcomes that were achieved by the testbeds include scientific publications, assignments, learning opportunities, testbed innovations, brochures with various solutions, and successful deliverables. Three testbed projects (TB_9,14,15_) were identified to accomplish the goal completely by becoming a regular line organization, creating national and international impact, or inspiring other institutions. All these three testbeds were supported by additional funding, which led to the sustainability of the testbed projects, further collaboration, and achieving the outcomes more than expected. Three projects were identified as 'still ongoing' (TB_4,6,12_). The sign (*) has been used to indicate the outcomes achieved by the testbed project.

## Discussion

Digital transformation has been one of the most prominent goals of Swedish society, aiming to reshape the economy and society itself.^37^ Financial investments have been initiated by the government, public sector, and individual municipalities and regional governments in Sweden to achieve this goal. Government agencies, such as Vinnova and the Swedish Research Council, have allocated financial resources to a range of parties, including private research sectors, to implement digitally innovated testbeds in different municipalities and regions across Sweden.^11^ The primary focus of the digital health testbeds in Sweden is to create a collaborative environment where health service and innovation can achieve their ultimate goal, i.e., improving the quality of care delivery.^11^

In this study, we sought to identify the characteristics of digital health testbed organizations in Sweden, how their stakeholders perceived the main benefits and challenges related to various testbeds projects, and how they adapted to overcome those challenges.

### Agents and diversity

Diversity is an essential characteristic of complex systems.^36^ In Sweden, digital health testbeds are diverse in terms of their organization, models of innovation, and the various stakeholders involved in the design and operation of the testbeds. For example, in Sweden, digital health testbeds are categorized either as labs or real or simulated testbeds. Also, the innovation model for stakeholders’ engagement was identified either as a triple helix, or quadruple helix model. The characteristics also included stakeholders’ perceptions and interpretations of the testbed definition, as well as their primary functions and purposes. Three main primary functions were identified: a testbed as a context (e.g. an infrastructure), a collaborative meeting place (e.g. an arena), and a technological testing place (e.g. an innovation hub).

Moreover, the definitions and concepts of testbeds are diverse. The definitions focus on the type of testbed and the discipline under which the testbed is designed or implemented. For example, a testing company may facilitate the system under the test and test framework needed to focus on healthcare. The development and maintenance of health information technology play a vital role in the facilitation of such systems. A study by the Swedish Agency of Growth Policy Analysis, suggested that implementation of the testbed projects was mainly determined by the organizations’ plans and operations, and managers’ understanding of the concept.^18^

Digital health testbeds in Sweden are designed to evaluate innovations for different kinds of care delivery, including person-centered care, social care, elderly care, and healthcare in general.^11^ Therefore, one of the most common purposes of the testbeds is the improvement of the quality of care delivery, in particular, in terms of safety, efficiency, and equity. Other purposes comprised developing, testing, or creating demand for e-health products and services, clinical applications, and technical innovation. The testbeds were also characterized by purposes that are not primarily care-related, such as building a collaborative, friendly climate or environment; developing and supporting regional and private business growth for small- and medium-sized companies; and creating a research environment and opportunities for education. According to our findings, these testbeds facilitate various goals, including jointly designing care services involving staff, patients, and relatives; developing user-driven innovation; and testing, evaluating, and improving clinical applications, products, and solutions. However, the primary focus of digital health testbeds in Sweden is to create a collaborative environment where health service and innovation can meet their ultimate goal, i.e., improving the quality of care delivery.

### Connections and communication: Key to successful collaboration

There was ample evidence of communication, connections, and collaboration between and among various actors throughout the design and implementation of the testbeds. These collaborations were either national, international, or both. Collaboration was further extended even to the academic level, such as through networking among doctoral students. For successful collaboration, stakeholder engagement is an important criterion. Transparency regarding who needs to be involved in designing and implementing the testbeds is essential. Entailing the right people at the right moment may impact the necessary analysis and the robustness of the analysis process.^38^ Therefore, the basic rules of e-health for solving clinical problems should involve a range of stakeholder engagement, including government structures, external consultants, large corporations, contractual arrangements, and even politicians.^3^

The key feature of a testbed program is tight collaboration, which should stimulate dialogue among key players, including healthcare organizations, academia, industries, government agencies, patients, potential customers, and other stakeholders sharing the goal of improving patient outcomes.^38^ Moreover, when value creation from the user perspective is in focus, the collaboration can be related to the model where the quadrupole helix is viewed as a network of relationships.^12^ A triple helix framework, originally consisting of academia, industry, and government can be developed towards a quadrupole helix if the involved actors combine their resources and engage in activities that are aimed to generate value for the fourth helix, civil society, and citizens.^12^ In a testbed program, if the shared goal of improved patient outcomes is achieved, this can create value for both end-users and the civil society as a whole.

The study by the Growth Analysis suggested that the implementation of previous Vinnova-funded testbeds was relatively easy and manageable when few stakeholders were involved. An abundance of stakeholders made coordination and administration more challenging and time-consuming.^18^

Within an organization, poor interactions between management and other actors involved in the testbed project resulted in frustration, delays in achieving the project goal, difficult working conditions, and a continuous need for dialogue. In large-scale testbeds, mobility management and content delivery have been the core challenges.^39^ Several participants reported that the distance between different testbed sites had been a challenge, hindering stakeholders’ involvement and product solutions from being found. This had been challenging, particularly for the laboratory environment-based (multiple) testbeds, which caused difficulties for research and documentation, as well as to-and-fro movement from one laboratory to another.

### Challenges due to uncertainty and unpredictability

Complex systems are intrinsically hazardous and unpredictable systems.^36^ A statement from the Growth Analysis reported that testbeds were failing to meet user needs and expectations due to various factors, including technological, organizational, and institutional factors.^18^ Many challenges during the testbed projects were associated with uncertainties about general focus and goals; the quality and future of healthcare; specific concepts and definitions; business and payment models; and legal aspects, rules, and regulations concerning testbed operation. The vague concept of the testbed is mentioned as one challenge, along with the excessive time used in a non-productive effort to define the testbed and its business model.

The findings of this study indicate a lack of clarity on the national level about the legal aspects of the testbeds, including legal guidance, the collection of agreement documents, and payment models. For example, testing innovations in the elderly care domain were a new experience, requiring lawyers to investigate applicable laws and regulations. The challenge of legal aspects was accompanied by an inability to achieve the project goals both in time and quantity. It is crucial to clarify various legal issues by, for example, identifying applicable laws, establishing what legally secure solutions should be in place, or determining how contractual aspects should be managed. Legal experts must address these questions to identify required actions, frame the appropriate guidelines, and recommend actions regarding new legal standards.^40^

### Challenges due to the busy healthcare environment

Healthcare is often populated by people in different roles, such as professionals and laypeople, as well as people of different ages, cultures, and backgrounds. Healthcare professionals perform daily under high pressure and with a heavy workload. Due to the imperatives of cultural safety and quality improvement in the healthcare environment, healthcare professionals also experience concerns regarding accountability and blame, mainly when under observation by visitors. Moreover, the vast majority of healthcare professionals are not personally interested in the design or implementation of technology use,^41^ which is also a source of frustration.^4,8,41^

A digital health testbed project in Sweden is often characterized by the involvement of different stakeholders, such as academia, industry, government, and the healthcare environment. Building new, innovative, and sustainable digital solutions for clinical decision support may be accompanied by data from the healthcare IT system.^12^ This may require research activities to test products or solutions in a busy hospital environment. Apart from day-to-day clinical routine practices, any extra activity, such as research, is subjected to compliance with various privacy and confidentiality regulations. This necessitates a formal review, informed consent, and ethics clearance before researchers perform their research activities in a busy hospital environment,^41,42^ particularly pertinent to technology application.^41^ Failure to follow these steps may potentially create a risk for disclosing personal or sensitive (identifiable) health information. It may also hinder carrying out regular healthcare activities, such as public health practice for healthcare professionals.^42^

This is consistent with our findings that one challenge for testbeds has been a busy healthcare environment. Despite the healthcare system's will to enable testbed research, obtaining healthcare operations and staff involved in product development was a hassle. Moreover, conducting a research study, i.e., collecting information and completing tasks, required ensuring privacy for those not participating in the research, and not causing any changes to the hospital's standard workflow. This required obtaining a healthcare access permit and informed consent, to be granted by the relevant authority, before conducting any study, which caused a delay in completing the testbed project.

### Challenges due to lack of adequate funding

According to our findings, inadequate funding has been one of the barriers to the design, implementation, and post-management of testbeds. The Growth Analysis identified a similar challenge regarding inadequate funding. Diverse sources of funding caused varying levels of complexity during the implementation. This further led to a shortage of professionals, which resulted in a heavy workload, including research, documentation, back-and-forth travel from one testbed site to another. Moreover, a lack of funding led to difficulty buying testing services, especially after implementing the testbeds.^18^

Testbeds that achieved all of the initial project's goals were well-funded before, during, and even after the implementation. Testbeds that executed most of the project's goals did not have adequate funding, or the budget was not extended after the implementation. This affected evaluating the implementation and testing the interoperability, resulting in few test operations being conducted.

### Adaptation and learning

A complex system is capable of responding to internal and external changes through adaptation and learning.^36^ In the presence of various challenges, the internal and external characteristics of the systems create defenses (a mechanism) against those challenges that, in turn, characterize the systems themselves. The defenses include technical components (e.g. technical details of the testbeds), and human components (e.g. facilitating commitment and trust among the stakeholders). Furthermore, various other components, such as organizational (organizational needs and entrepreneurial ideas), regulatory (policy and guidelines), and environment-related (lack of access to healthcare sites) aspects, are included. Humans function as both producers and defenders against failure, i.e., humans diligently adapt the system to maximize production and minimize challenges. Human expertise in the complex system changes according to any technology-oriented changes, or in response to a new need that arises in the system. For example, this may cause new expertise to be recruited, hired, or replaced.^43^

No matter what changes are introduced, whether human or technological, the possibility of new forms of problems remains.^43^ For example, a solution, i.e., shortening the length of the technical description of the test, was further supplemented by a stand-alone test script so that it was possible to run the test on their own site before connecting to the testbed. This additionally led to better implementation and fewer complications in the later stages of interoperability testing. On the other hand, novel and unpredictable situations can also lead to innovation and progress because it is in the nature of the human and complex systems to adapt to challenges. For instance, new experience requiring the lawyers to investigate what laws and regulations the activity needed to conform to testing innovations in the elderly care domain resulted in building up a structured operation for testing the innovations and achieving the project goal by a good margin.

### The importance of history

The initial conditions and the development of complex healthcare systems are important to recognize when designing and implementing digital health testbeds.^36^

Over the last three decades, healthcare quality has increasingly been on the agenda, because the healthcare system can itself cause harm to patients by offering inappropriate care or delayed care, or when the care provided fails to work properly.^3^ Moreover, care provided should be satisfactory concerning the dimensions of quality of care, such as accessibility, appropriateness, safety, efficacy, effectiveness, timeliness, patient-centeredness, equality, and equity.^44,45^ One or more missing care aspects entails the incomplete quality of care. For instance, providing inappropriate or ineffective care should be unacceptable, even if that care is safe and delivered on time.^46^ Therefore, a holistic view incorporating all dimensions of quality of care must be adopted in developing, testing, and creating demand for e-health products, solutions, and services. This may also include legal and ethical aspects, as well as the benefit and effect of such technologies for patients, healthcare professionals, and healthcare organizations as a whole.

The best ideas for innovation should come from the people who understand the conditions of healthcare, and have participated in developing healthcare systems.^47^ For example, suppose healthcare is considered from the perspective of user value. In that case, the healthcare system must integrate resources to treat the value as patient resources, and create the network around the patients that will create further resources. In this case, doctors, nurses, and other healthcare professionals need to build a network around patients, and integrate resources.

The purpose of the testbed design should be to understand users’ needs and the value created around these needs. For example, one has to know how an ‘e-health application' creates value in people's lives, and to change the service system behind such an application to suit the need and value created. This necessitates identifying how innovations work, re-building innovations, rearranging procurement, and managing the organization.

### Implications for practice

Based on considerations arising from the literature, government reports, and findings from this study, the following implications for improvement in practice are suggested when designing a framework for digital health testbeds. These may help overcome new, unforeseen, and unpredictable challenges that are often encountered by a testbed program.

- **Ensure the quantity and quality of the stakeholders from the beginning**: Collaboration is the key to success for any testbed program. According to the testbeds’ needs, a range of stakeholders may be considered, including academics, researchers, industry experts, system developers, or government agents. Involving the correct number of people at the right time may bring the maximum value for successfully achieving the ultimate goal of testbeds implementation.
- **Identify the factors that need to be clarified to overcome uncertainty:** These include identifying the key activities and setting up a clear focus, scope, business model, and management resources. This may begin with clarifying some uncertainties identified in the study, for example, the project goal, definition, and concept of the testbeds, as well as the business model, strategy, or method used for design and implementation purposes. Some ameliorating steps can be carried out in response to some of the issues identified in this study, such as setting up legal aspects – contractual agreements and documentation, payment models, and applying for access to any (hospital) site for research purposes. The nature and scope of the intervention(s) to be implemented should also be clarified. If the technology to be introduced into the clinical pathways is to be mapped out appropriately, it may function for evaluation design and adjust the working pattern if necessary. For example, data sharing is of core importance in most current digital health testbeds, which must be carried out in full compliance with the General Data Protection Regulation.
- **Commission an evaluation for better testbed management:** Assigning an evaluation at-and-from an early stage makes it possible to continuously test and verify the testbed implementation, possibly reducing costs and preventing frustration in later phases. This can be done by ensuring a fair and open procurement process. For example, appointing an evaluation team as a suitably skilled, external, and independent party may help obtain advice on several improvement factors, including contractual arrangements, legal guidance, and payment models. Both the testbed members and evaluation teams should work collaboratively so that the workflow is effective and efficient on daily routines to ensure individual responsibilities, expected risks, and mitigation measures. Besides, customers should evaluate the testbed products and services and check if their needs have been met.
- **Plan for unexpected challenges to mitigate risks of uncertainty:** This should identify what is going wrong during the testbed implementation and respond to any specific problem occurring at any stage quickly and efficiently. For example, one of the contingency plans may be enhancing the availability of experts, such as communication strategists or coordinators. Such plans should maintain a steady testbed process, with things going right throughout the design and implementation process.
- **Ensure adequate funding for the sustainability of the testbed project**: For long-term sustainability, continuous funding should be ensured, including a grant-funding program supporting new and unforeseen challenges that may arise during or after the testbeds’ design and implementation. As an illustration, an e-health based testbed focusing on health information technology and data sharing should foresee data interoperability and infrastructure and data regulation challenges. If these issues remain unresolved due to inadequate funding, the system may fail and block further development. Any form of funding, including independent, public, or private, internal or external, should be distinguished and utilized in appropriate phases of the testbed implementation, such as research, evaluation, and product development. This will ensure the efficient and effective use of resources and help overcome the challenges before, during, and after the testbed implementation.
- **Disseminate the lessons learned and share the insights gained**: Summarizing and sharing the practical challenges, mitigation, and prevention measures, as well as the organizational or economic impact, at each testbed developmental phase, whether early, developmental, or late-stage, is the ideal way to move forward. Lessons learned from testbed implementation findings should be shared with all possible partners and stakeholders (internal and external) in the form of written documents and presentations at national and international events, meetings, or conferences. Dissemination may include various events, including seminars, workshops, discussions, and scientific publications.

## Strengths and limitations of this study

This study was conducted during the COVID-19 pandemic, which has been assumed to be the reason for low response rates. The interviews were conducted either through written response, or audio-conferencing by the principal investigator alone. Participants who agreed to participate through audio-conferencing seemed to have more professional experience, and provided more detailed information with thoughtful insights. Like other qualitative exploratory studies, the data set was limited to subjective impressions, sub-stranded personal bias, and participants’ content knowledge.^8^ Other limitations included a small sample size, a lack of participant motivation for more detailed conversation, and the researchers’ susceptibility to bias in the interview questions.

Thematic analysis with a deductive approach has been helpful in interpreting the information (narrative texts) collected through interviews.^48^ Extracting the lists themes further strengthened the analyses to provide detailed information. Nevertheless, the analysis may have been prone to the researchers’ bias (subjective) to interpretation, and inherently reductive,^49^ focusing on words or phrases, especially in the case of complex texts.

In addition, the results of the thematic analysis, such as whether a project meets its purpose, need to be treated with caution. Such outcomes are difficult to measure. For example, the average time for a medical device to reach market can be as long as seven years. In addition, it is difficult to demonstrate the impact of a particular company or product to the external stakeholders, as they meet only for a brief moment during product development.

## Conclusion

This study provided insights into different testbeds’ functions and their various factors, such as facilitating, challenging, and ameliorating factors. Viewing the testbeds’ organizations under the norm of complexity science (complexity theory and CAS) offered explanations about how various aspects of the testbeds were challenged, and how they adapted to overcome and improve system issues. Despite the different testbed definitions and concepts used by multiple stakeholders, collaboration, connections, and communications among the stakeholders were the key to achieving the ultimate goal. The complex nature of testbeds makes their design and implementation more susceptible to various challenges. Insufficient access to the healthcare environment, legal aspects, and inadequate funding have been obstacles in designing and implementing the testbeds and accomplishing their purposes. Even these challenges may or may not have a direct impact. They often cause delays and inconvenience regular workflows, creating frustration among the stakeholders, system ineffectiveness, and inefficiencies. Several adaptations or ameliorating factors, such as sharing and creating learning opportunities, technical implementation, and commercialization of e-health solutions, ensured the progress and sustainability of the testbeds. Identifying the stakeholders and relevant factors, commissioning an evaluation, backing up with a contingency plan, securing adequate funding, and disseminating findings can help overcome the unexpected and unforeseen challenges, and improve the testbeds’ design and implementation.

## Supplemental Material

sj-docx-1-dhj-10.1177_20552076221075194 - Supplemental material for Digital Health Testbeds in Sweden: An exploratory study Click here for additional data file. Supplemental material, sj-docx-1-dhj-10.1177_20552076221075194 for Digital Health Testbeds in Sweden: An exploratory study by Md Shafiqur Rahman Jabin, Evalill Nilsson, Anna-Lena Nilsson, Patrick Bergman and Päivi Jokela in Digital Health

sj-docx-2-dhj-10.1177_20552076221075194 - Supplemental material for Digital Health Testbeds in Sweden: An exploratory study Click here for additional data file. Supplemental material, sj-docx-2-dhj-10.1177_20552076221075194 for Digital Health Testbeds in Sweden: An exploratory study by Md Shafiqur Rahman Jabin, Evalill Nilsson, Anna-Lena Nilsson, Patrick Bergman and Päivi Jokela in Digital Health
