# Supplementary material for: Digital Health Testbeds in Sweden: An exploratory study
Source: Digit Health. 2022 Feb 14;8:20552076221075194. doi: 10.1177/20552076221075194 (PMC8848084; doi:10.1177/20552076221075194)
Supplement: sj-docx-1-dhj-10.1177_20552076221075194 - Supplemental material for Digital Health Testbeds in Sweden: An exploratory study [file sj-docx-1-dhj-10.1177_20552076221075194.docx]

# Appendix A

| \| **No.** \| **Phrases or keywords** \| \| --- \| --- \| \| 1 \| Radiology departments can handle more patients with the same number of staff \| \| 2 \| Improve diagnosis, patient safety and quality of care \| \| 3 \| A specifically developed Light Field display \| \| 4 \| Software for rendering 3D visualizations from MRI images \| \| 5 \| The same software was adjusted to present corresponding visualizations \| \| 6 \| A stereo display and a standard display \| \| 7 \| A company called “S” (no longer existing) had developed this test bed \| \| 8 \| M researched on theoretical aspects on the rendering and visual reproduction \| \| 9 \| The company S did all technology implementation \| \| 10 \| Employed theory on multiple view geometry and visual perception. \| \| 11 \| “M” had no possibility to control details in the testbed construction \| \| 12 \| To have full control of those new testbeds in later research \| \| 13 \| Developed the testbed ourselves \| \| 14 \| The testbed resulted in several scientific results \| \| 15 \| More results than anticipated \| \| 16 \| An arena for creating, testing and showing the solutions \| \| 17 \| Focus on IoT and urban mobility solutions \| \| 18 \| Provide testbeds with a wide array of digital infrastructure available \| \| 19 \| Applications and devices \| \| 20 \| We bring together academia, public sector and business \| \| 21 \| No methodology in the beginning \| \| 22 \| A couple of principles and strategies \| \| 23 \| Open collaboration is the key \| \| 24 \| “Win-win” situation \| \| 25 \| Not owning any of the infrastructure that we offer in our testbed \| \| 26 \| Plug-and-play playground \| \| 27 \| Different actors provide what they have and share with each other \| \| 28 \| A big digital playground \| \| 29 \| A kitchen where you can meet chefs, look at recipes \| \| 30 \| Not own anything while offering the latest technology \| \| 31 \| Infrastructures provided by our partners \| \| 32 \| Agreements for this must be signed \| \| 33 \| Contacts, project management, technology management, communications etc \| \| 34 \| Very few tests have been conducting using the testbed \| \| 35 \| Companies and other stakeholders were unsure as for what rules apply \| \| 36 \| Internal re-organization \| \| 37 \| Taste new dishes and compose new ones together \| \| 38 \| Choose a couple of cases to focus on \| \| 39 \| The players have an idea what we are working with \| \| 40 \| What is possible to work on together with other players with similar interests \| \| 41 \| Meeting place and collaboration is the key to any testbed \| \| 42 \| Offer the infrastructure without the tools, matchmaking, facilitating etc. \| \| 43 \| More than a testbed – a collaboration arena \| \| 44 \| Set up a meeting place and help out with the whole process from matchmaking \| \| 45 \| Sharing lessons learned with other players nationally and internationally \| \| 46 \| Allow for scalable solutions. \| \| 47 \| We have a long way to go \| \| 48 \| Testbeds are all integrated in daily routines \| \| 49 \| Improve efficiency and effectiveness of elderly care digital solutions \| \| 50 \| Create growth for small and medium sized companies \| \| 51 \| Welfare technology targeted for elderly care \| \| 52 \| Chose not to institutionalize them but rather re-create them for each purpose \| \| 53 \| Test pre-market solutions \| \| 54 \| The solution is not designed with our institutionalized needs but the elderly in mind \| \| 55 \| The testbed must adapt to some degree to the entrepreneurs line of thought \| \| 56 \| Each testbed is governed by the existing line organization in the respective municipality \| \| 57 \| Testbeds are all integrated in daily routines \| \| 58 \| Chose not to institutionalize them but rather re-create them for each purpose \| \| 59 \| Constantly re-gain commitment from top management \| \| 60 \| Matching entrepreneurial ideas and solutions with organizational needs \| \| 61 \| Openness to new ideas within the mother organization \| \| 62 \| Time and resource consuming process but also a major contributor of learnings \| \| 63 \| A major contributor of learnings. \| \| 64 \| Maintaining a steady flow of entrepreneurial ideas to match \| \| 65 \| The test beds contributed to us reaching the project goals \| \| 66 \| Created lots of learning opportunities for all stakeholders \| \| 67 \| We have used the definition set by “V”straight off \| \| 68 \| It is not an exact science but rather something to aim for \| \| 69 \| Knowledge building \| \| 70 \| Driving healthcare quality through research & education \| \| 71 \| The collaboration was initially formed by the City of “G” and “C”, University of “C”, and “R”. \| \| 72 \| Over time we have involved the science parks as well as the Region “G” \| \| 73 \| Among the 50-60 testbeds in the region we have several private initiatives \| \| 74 \| Many testbeds involve companies in their setup \| \| 75 \| Agenda 2030 in focus \| \| 76 \| Speed up commercialisation and also aim for longevity for testbeds \| \| 77 \| Make them last over time with sustainable value propositions \| \| 78 \| Testbeds lasting only through the financed project time 2-3 years \| \| 79 \| Lacking commercial industry focus \| \| 80 \| The question needs to be put to each individual testbed \| \| 81 \| We don’t use a formal definition of a testbed \| \| 82 \| We open up our business operation for outsiders to perform trials. \| \| 83 \| Normally the management in the appointed department needs to be involved \| \| 84 \| It impacts the daily operation \| \| 85 \| Create research environment \| \| 86 \| Internet of things (IoT) \| \| 87 \| Collaboration with the city and companies within the region \| \| 88 \| Have a “testbed” called H Works, which is testing new \| \| 89 \| New methodologies in terms of how to develop innovation initiatives \| \| 90 \| Lean start-up, agile development teams, Google design sprint, service design etc. \| \| 91 \| Proven successful methods in the private sector in large organisations \| \| 92 \| Establishing new operations like a testbed demand a lot of communication \| \| 93 \| The foundation must be to create trust with businesses, and their involvement \| \| 94 \| The testbed will be a side-track with small probability of implementation \| \| 95 \| There must be a buy in from higher management, and a key stakeholder \| \| 96 \| People´s time is the major bottle neck \| \| 97 \| We are in the middle of this project \| \| 98 \| A number of successful deliverables \| \| 99 \| Empower colleagues within the organization \| \| 100 \| New experiences and tools for future project \| \| 101 \| The project offers companies a complete test infrastructure \| \| 102 \| Meeting places and collaborative environments \| \| 103 \| The test infrastructure is composed of 2.5 testbeds in elderly homes, health centres \| \| 104 \| Building test environments with unique and innovative 5G and edge functionality \| \| 105 \| Indoor coverage where a central orchestration function dynamically distributes software to the local test environments \| \| 106 \| The focus is on elderly housing \| \| 107 \| Region's health centre together with the municipality's unit \| \| 108 \| Run the tests in one or more testbeds \| \| 109 \| Improving the quality of healthcare & elderly care \| \| 110 \| The project focuses on SMEs and other companies in “U” \| \| 111 \| Deliver products and services for healthcare enabled by 5G connection or creating innovation in wireless communication \| \| 112 \| Involves industry experts in wireless communication \| \| 113 \| Other expert support is hired when needed (e.g. technical analysis, market analysis) \| \| 114 \| Stakeholders: “LT”, “UT”, “L” science park, “S” science city, “UI”, “ABI”, “LTU”, “R”, “L” municipality, “O” municipality, “S” municipality, Region “N”, “EU” \| \| 115 \| Develop integration towards an health information architecture standard \| \| 116 \| A testing company \| \| 117 \| Focus on healthcare and the development and maintenance of the IT-systems \| \| 118 \| The testbeds usually consist of the System Under Test (SUT) \| \| 119 \| The test tool Selenium where you write your test suites \| \| 120 \| Services that meet or exceed customer's and other stakeholders' expectations \| \| 121 \| Improve the quality of deliveries through upgraded working method \| \| 122 \| Rapid set-up of test environments \| \| 123 \| The stakeholders are usually Regions, healthcare system developers, governments, and sometimes research (industry/university) \| \| 124 \| We have a test philosophy and test methodology that we make use of as a base \| \| 125 \| We make use of the experience of our employees to setup the solutions \| \| 126 \| Do market surveys if we need new technology or new approaches. \| \| 127 \| From experience have seen this successful \| \| 128 \| Context approach \| \| 129 \| Analyse the given context before we plan our testing effort \| \| 130 \| Our own experience \| \| 131 \| Technology prevents us from finding solutions \| \| 132 \| The cost to implement the solution is too high \| \| 133 \| Distance to the stakeholders has given us some challenges \| \| 134 \| Mount a camera so the physical testbed can be seen from distance \| \| 135 \| The stakeholders usually read a technical description to implement the solution(s) \| \| 136 \| This usually took a long time and when they were ready to connect to the infrastructure \| \| 137 \| Test their implementation \| \| 138 \| Test and verify the implementation which saves cost and frustration \| \| 139 \| Standalone test scripts to run on their own site before connecting to the testbed \| \| 140 \| Better implementation and less complications \| \| 141 \| Interoperability testing \| \| 142 \| A meeting place/venue where innovators can meet the intended users of the innovation \| \| 143 \| Create customized meetings and tests with experts (users) \| \| 144 \| The testbed is a business where innovators turn to connect with users \| \| 145 \| Within the testbed projects, innovators, health care professionals and the elderly and/or persons with disabilities are involved \| \| 146 \| We had 16 partners (health care- and care providers, the county council, business support organizations, the academia and pensioner's organizations) \| \| 147 \| Today, we work closely to several of these organizations \| \| 148 \| Involve those who should be concerned \| \| 149 \| We involved all of our partners in the design of the testbed \| \| 150 \| No discussion regarding service design \| \| 151 \| But, in retrospect we can see that it was that very method we were using \| \| 152 \| The method had previously been used in the work on welfare technology in our municipality \| \| 153 \| Also proving to be a success factor \| \| 154 \| An on-going evaluation researcher followed up how the business was experienced by innovators and test participants (elderly, staff) \| \| 155 \| Gave us guidance during the establishment. \| \| 156 \| Creating the testbed was the legal guidance and the collection of agreement documents \| \| 157 \| Testing innovations in the elderly care domain was a new experience \| \| 158 \| Requiring the lawyers investigating what laws and regulations were needed \| \| 159 \| Develop a payment model \| \| 160 \| Politicians in the municipality considered the testbed business as important to finance the activity \| \| 161 \| Build up a structured operation for testing of innovations within context of elderly and persons with disabilities \| \| 162 \| The number of innovations we would test during this period \| \| 163 \| By the time the testbed went into a permanent proceeding, the main goal was achieved \| \| 164 \| The number of tested innovations as one of the goals, was achieved by a good margin \| \| 165 \| Another aim was to produce the legal guidance, which was also achieved. \| \| 166 \| To what extent innovators needs have been met that we mainly evaluate \| \| 167 \| Follow-up researcher's reports and through evaluation questionnaires \| \| 168 \| We submit to the innovators after the service has been completed \| \| 169 \| They are very satisfied with our delivery \| \| 170 \| We used the “V” definition \| \| 171 \| A physical or virtual environment \| \| 172 \| Companies could develop, test, and implement new products, facilities, processes or organizational solutions \| \| 173 \| Collaboration with actors within the healthcare sector or the elderly care \| \| 174 \| Management of sustainable, strategic and effective innovations \| \| 175 \| Support healthcare & elderly care for testing & developing new solutions \| \| 176 \| We did not have a well-defined method \| \| 177 \| We based the work on external monitoring to a large extent \| \| 178 \| Including the needs of our clinics and researchers, and business \| \| 179 \| Need to evaluate usability \| \| 180 \| Build physical test bed environments (care environments) \| \| 181 \| Created a virtual IT test bed environment to test systems under development \| \| 182 \| Difficult to reach out, to become known, both internally in a large organization and within business and the academia \| \| 183 \| Test beds were very unknown concepts in health care \| \| 184 \| Anchorage was required for decision-makers at different levels to understand what it meant and what benefits it could generate \| \| 185 \| A lot of work to explain the difference between innovation and continuous improvement \| \| 186 \| Explain how they complemented each other \| \| 187 \| We went from project to regular line organization \| \| 188 \| The project goals were achieved \| \| 189 \| Ensure further funding \| \| 190 \| We used the “V” definition of a test bed \| \| 191 \| A physical or virtual environment \| \| 192 \| Companies, academia and other organizations can collaborate \| \| 193 \| Development, testing and introduction of new products, services, processes or organizational solutions in selected areas \| \| 194 \| Improving quality of care elderly & disabled people \| \| 195 \| Increase adoption of new technology with understanding of new solutions \| \| 196 \| Understanding of new solutions \| \| 197 \| Those who ran the test bed were “AN” science park, “O” Municipality, “O” University and Region County \| \| 198 \| User centred development through Double Diamond design process \| \| 199 \| Methodical information need analysis (MINA) – a municipal wide model for inventory \| \| 200 \| Defining and analysing needs. \| \| 201 \| Kano model is central to the work of developing a solution and/or innovation \| \| 202 \| Needs assessment \| \| 203 \| Many parties which became an added value \| \| 204 \| Challenges in getting all parties involved \| \| 205 \| We put quite a lot of effort into was business model and offer to the companies \| \| 206 \| Should the test bed be useful for a fee, and if so, at what level? \| \| 207 \| Tested products/innovations but then they did not make it any further \| \| 208 \| Implementation and wider practical usage \| \| 209 \| One lesson we have learned is to base ourselves on the needs of the user/business \| \| 210 \| Legal aspects \| \| 211 \| Various supervision solutions \| \| 212 \| This is not clear from the national level \| \| 213 \| We believe it will be easier with the report made by “X” \| \| 214 \| Demographic challenge \| \| 215 \| We achieved most of our goals \| \| 216 \| We received funding from “V” \| \| 217 \| We have continued working with the test bed, so it is a good result \| \| 218 \| Partly taken from “V”(definition) \| \| 219 \| A virtual environment \| \| 220 \| Companies, academia, other organizations and need owners \| \| 221 \| The test bed provides expertise, networking, and physical testing environments \| \| 222 \| Support private business offering healthcare services \| \| 223 \| Testbed business does not devote itself to clinical trials/research this year \| \| 224 \| FS is responsible for those parts, whom “X” cooperates with when required \| \| 225 \| Our test bed was started as part of “CT” \| \| 226 \| Built according to the conclusions drawn during the “V” funded pilot study \| \| 227 \| A review and analysis was made of the conditions that existed in “S” \| \| 228 \| Inspiration and examples from the rest of the world were reviewed \| \| 229 \| Study visits were made \| \| 230 \| Work together with the healthcare services to create value for patients and employees \| \| 231 \| The basic idea has been further developed through participation in other test bed projects \| \| 232 \| A Nordic test bed collaboration and improvement was explored \| \| 233 \| Improved through participation in the Baltic Sea project \| \| 234 \| A large number of Living Lab collaborated to improve their own operations \| \| 235 \| Develop a transnational collaboration proposal \| \| 236 \| A number of other collaborations and test bed initiatives \| \| 237 \| Primarily in health, but also in one of our other innovation areas \| \| 238 \| The most difficult part is to maintain a sustainable testbed environment over time \| \| 239 \| Difficult to get health care operations and staff involved in product development \| \| 240 \| Lack of time in health care for everything except the basic assignment \| \| 241 \| There is often the will from within the health care system \| \| 242 \| Test beds are generally project dependent to a great extent \| \| 243 \| Difficult to retain the right staff and expertise \| \| 244 \| A key to success is that there is a Living Lab / test bed coordinator \| \| 245 \| Got attention at the “X” conference in Athens 2019 \| \| 246 \| Have partial financing from the Region \| \| 247 \| Must seek money and run projects to gain sustainability \| \| 248 \| Handles both external companies' requests for support and assistance, and employees' ideas from the health care sector \| \| 249 \| The goals have previously been "vague" \| \| 250 \| A few years ago we started to measure the number of qualified \| \| 251 \| Whether the help we give the companies, actually has a decisive importance for them \| \| 252 \| We achieve our goals when helping companies navigate and by finding test opportunities \| \| 253 \| H people behind the ideas in their work \| \| 254 \| Difficult to measure how important our impact was \| \| 255 \| An average time for a medical device to reach the market can be as long as seven years \| \| 256 \| Difficult to demonstrate our impact on this particular company or product \| \| 257 \| Companies usually come to solve a problem or take the product one step further \| \| 258 \| We only see them for a brief moment in their product development \| \| 259 \| Develop digital solutions increasing individuals capability & quality of life \| \| 260 \| Ensure equal health, business growth & efficiency in public health services \| \| 261 \| Quadruple helix methodology \| \| 262 \| Small and medium sized companies \| \| 263 \| We have different stakeholders for different challenges and innovation cases \| \| 264 \| Sometimes public sector, sometimes individual contractor/ entrepreneur \| \| 265 \| Service design \| \| 266 \| Double diamond methodology \| \| 267 \| User involvement to really ensure that the end product is usable and based on the needs \| \| 268 \| To be allowed to document the environment and testing on film \| \| 269 \| Collaborate to develop test new products, services, processes or organizational solutions \| \| 270 \| The research team was luckily granted this \| \| 271 \| To see the effects of using digital tools to guide the visitors and patients at the hospital \| \| 272 \| A busy environment \| \| 273 \| Collect the information and completing the work required, without disturbing people, \| \| 274 \| Ensuring privacy for those not taking part in the study \| \| 275 \| Not causing any changes to the normal flow at the hospital \| \| 276 \| The case team faced similar challenges \| \| 277 \| As the study by “CTF” isn’t complete yet \| \| 278 \| We cannot answer the first question for them as yet \| \| 279 \| To ensure the researchers could access the data they needed \| \| 280 \| Depending on the outcome, this could result in better, user centred mobile applications \| \| 281 \| Great learnings and insights were gained with testbed in hospital environments \| \| 282 \| Developing relevant documents and a better understanding for the legal aspects \| \| 283 \| This could be beneficial for people and businesses in need of test beds. \| \| 284 \| An arena to be defined as a node with different functions. \| \| 285 \| The test bed will therefore not have a specific physical infrastructure \| \| 286 \| The solutions evaluated in the test bed will be disposed at the real needs owners \| \| 287 \| The concept for our test bed is to match the real needs of the owners with solutions \| \| 288 \| How well the product meet the needs of the owner \| \| 289 \| Contribute to product development and innovation \| \| 290 \| To increase the use of solutions in the dense city to a greater extent \| \| 291 \| To create increased demand and contribute to the development of new solutions \| \| 292 \| Community builders test different types of green-blue solutions \| \| 293 \| The solutions (techniques and methods) are evaluated by independent experts \| \| 294 \| The testbed will serve as a node for solutions by offering a range of other functions \| \| 295 \| Offer courses and training, counselling and expert help, marketing and networking. \| \| 296 \| We also work with a reference group \| \| 297 \| Partners: ”M”, ”MS”, ”SB” Hub, ”SLU”, ”IVL”, ”M” Institute, ”S” Institute, ”M” Kommun \| \| 298 \| Build a consortium that will remain after the end of the project \| \| 299 \| Solutions will be tested by the real needs owners \| \| 300 \| Can find the right manufacturer for the right needs through its wide network \| \| 301 \| Product selection to product evaluation \| \| 302 \| The package is customized for each new customer \| \| 303 \| Business Model Canvas \| \| 304 \| Spent a lot of time defining and redefining what our test bed is and what kind \| \| 305 \| What kind of value proposition is needed to make it a sustainable business model \| \| 306 \| The project is ongoing \| \| 307 \| A number of seminars based on needs they have expressed \| \| 308 \| A brochure with nine different types of solutions \| \| 309 \| The test bed will be of great benefit within a broad front \| \| 310 \| I don't think we used one (definition) \| \| 311 \| A place where you can try out or open up other companies and organisation to test out a lot of problems and healthcare organisations \| \| 312 \| We never had the “V”funding that we have running through 2003 \| \| 313 \| Sort of hug along with the network of others \| \| 314 \| It's a very diverse definition, how we understand, what the testbed is \| \| 315 \| “Innovation hub" or may be, "innovation system” \| \| 316 \| There was a group of innovations people but have got funding from “V” \| \| 317 \| I travel, with them in different places, we were through all of those meetings \| \| 318 \| “V”forced their testbed program \| \| 319 \| We sort had an idea what we wanted to do \| \| 320 \| “V”program for testbeds and we applied and got funding in the program in 2013 \| \| 321 \| Application hub \| \| 322 \| (Our) lab is always explorative \| \| 323 \| Have research organisation and also interested in connecting it \| \| 324 \| The backbone is service logics \| \| 325 \| Use them and involve those management people and healthcare through a lands of service logics \| \| 326 \| X university service centre \| \| 327 \| 80-90 people from service environment \| \| 328 \| A global actor in that field \| \| 329 \| A different eye that your value is created \| \| 330 \| We are in a big shift \| \| 331 \| A more industrialised product logic system \| \| 332 \| Consumer or patient \| \| 333 \| Someone consumes that value and that's why we call it service logics \| \| 334 \| Involves services because healthcare is a service \| \| 335 \| Health is not created by a doctor but it is created by you. \| \| 336 \| Health is not in their life, it's your life \| \| 337 \| Wanted to shift that to see if we look through healthcare from a service logic perspective \| \| 338 \| Health is different for different people \| \| 339 \| understand something around resource integration \| \| 340 \| healthcare is a mix of patient resources, relative resources \| \| 341 \| The network you have around the patients will create resources \| \| 342 \| Sometimes depending on the situation of course you will need the resources \| \| 343 \| eHealth conferences \| \| 344 \| Invitation from couple of countries \| \| 345 \| The product logic system \| \| 346 \| How we produce our services and products in healthcare \| \| 347 \| Research and the universities around us \| \| 348 \| Course design \| \| 349 \| Design is purposely understand your user's need \| \| 350 \| The value creation work with healthcare because we are part of healthcare \| \| 351 \| National person centred care for instance \| \| 352 \| Need to innovate services including culture which is person-centred \| \| 353 \| Clinic centre for innovation \| \| 354 \| Experience based design \| \| 355 \| Discussion with mind labs and other design driven labs \| \| 356 \| Framed it much more accustomed \| \| 357 \| A growing design lab movement around on the globe, rather than a testbed thing \| \| 358 \| A share or an architecture or an eHealth application building apps \| \| 359 \| A meeting with the doctor \| \| 360 \| Created an environment \| \| 361 \| eHealth is much broader than \| \| 362 \| What it creates value in people's life \| \| 363 \| Change the service system behind that app \| \| 364 \| It actually happens in the organisation how we take care of \| \| 365 \| Rearrange procurement, management and other thing \| \| 366 \| A service logic perspective \| \| 367 \| Design a new service, including also how we produce it \| \| 368 \| I think there is a challenge every day to build innovation capacity within healthcare \| \| 369 \| Healthcare is a challenge \| \| 370 \| Corona thing changed it \| \| 371 \| “V” money received \| \| 372 \| University and the national research collaboration \| \| 373 \| Connected national design \| \| 374 \| A huge success and that was also made part of the problem \| \| 375 \| A design embedded lab in Swedish public system and definitely within healthcare \| \| 376 \| Build capacity within the county council \| \| 377 \| National projects and collaborations in running \| \| 378 \| Managing two out of twenty H20 financed project within Sweden \| \| 379 \| One was more about digitalisation and the other was more PhD training network platform \| \| 380 \| Radical dilemma \| \| 381 \| It's a balance between inside and outside \| \| 382 \| Sometimes we get too much outside \| \| 383 \| That was super tiring and very hard time \| \| 384 \| Have some freedom from being working in lab way \| \| 385 \| Run projects not on purpose to make mistakes \| \| 386 \| Drive research a learning and understanding from wide (perspective) \| \| 387 \| The results and the learning point from the organisation (to) build a lab \| \| 388 \| You create a space in the organisation where you are allowed to fail \| \| 389 \| Patient safety and daily improvement, quality. \| \| 390 \| Hinder us from what can healthcare be in the future \| \| 391 \| I think that's a big huge point for having a lab \| \| 392 \| Employ service designer \| \| 393 \| We thought that there is not a need for many labs in Sweden \| \| 394 \| Logic designs user participants agency and capacity \| \| 395 \| The collaboration is set up by a letter of intent and formal agreement \| \| 396 \| I facilitate that national movement and collaboration \| \| 397 \| Colleagues from travelling to and from the region \| \| 398 \| A big turning point where opted not to build an organisation \| \| 399 \| Achieved for person-centred care \| \| 400 \| A big success for us \| \| 401 \| National and international impact than we thought \| \| 402 \| We came much further than we thought \| \| 403 \| County council and municipality want to get on board \| \| 404 \| Applied for fifteen PhD strong trainers and training network \| \| 405 \| A bit of management staff that you get into a crisis like Corona \| \| 406 \| Reorganise or rebuild a bit stronger facilitation process \| \| 407 \| Grow up with national partnership \| \| 408 \| An informal research group write application together \| \| 409 \| Researcher who are interested in person centred care, design, innovation, and service logics with 14 people from seven universities, mainly within Sweden and Norway \| \| 410 \| Collaborated a work in H laboratory which is sort of a infrastructure of the lab \| \| 411 \| An infrastructure where all expert people focus in speech and languages \| \| 412 \| A psychologist, cognitive in neuroscience \| \| 413 \| I am an assistant professor as Swedish as a second language \| \| 414 \| I am used to having a lab \| \| 415 \| A psychologist \| \| 416 \| Some experiments do studies, in a different way than they have done so far \| \| 417 \| A scientist \| \| 418 \| People mostly do text analysis, another type of research. \| \| 419 \| We said we need to have a lab where we can do this type of research \| \| 420 \| Introduce it other people at the institution and also go broad \| \| 421 \| Making it more possible for us do interdisciplinary work too. \| \| 422 \| I would call it as sort of infrastructure or a lab \| \| 423 \| It should be affordable for such a study \| \| 424 \| Type of grant and equipment to do that type of research \| \| 425 \| Handle these microphones \| \| 426 \| Software program \| \| 427 \| Eye tracker \| \| 428 \| Workshops \| \| 429 \| A lot of support from the scientist, also the institution, and the prefect, with vice prefect \| \| 430 \| The experiences that I have from the universities \| \| 431 \| The humanity's lab in a way \| \| 432 \| I can get more people from our department to come and do research with us \| \| 433 \| Do with all those equipment \| \| 434 \| Giving demos \| \| 435 \| People contacting us to visit the lab \| \| 436 \| We are taking students into the lab and student taking method courses \| \| 437 \| The type of research and they can come to have a sort of ideas \| \| 438 \| Use some of our equipment to study the questions they are interested in \| \| 439 \| Doing the education and trying to get people in there \| \| 440 \| We have four students doing Bachelor thesis and sort of writing and studying \| \| 441 \| You are interested in EEG \| \| 442 \| Set up a workshop towards your need \| \| 443 \| How do I stimuli presentation or having a course in EEG, a course in ERP \| \| 444 \| Eye tracking or in reaction time \| \| 445 \| They should be able to start collecting the data \| \| 446 \| We help you make changes and make pilots to help your studies \| \| 447 \| Develop sort of scripts and also sort of PDF \| \| 448 \| Special manner in a sort of collect data \| \| 449 \| Similar approach because then it was easier to support them \| \| 450 \| A small group, and small scale \| \| 451 \| I don't know that type of action research method \| \| 452 \| I used my experience, this worked here and that was my goal \| \| 453 \| We had a lot of Post-docs in the lab, PhD students \| \| 454 \| Peer-learning \| \| 455 \| A small workshops where we just looked at each other’s data \| \| 456 \| I look at your EEG data that looks crap \| \| 457 \| I don’t want to show my data because I use a lot filters \| \| 458 \| There was sort of milestone for having these collaborations \| \| 459 \| Changing the culture to something that I was used to \| \| 460 \| We are applying for grants and funding for a lab \| \| 461 \| We have to find a room for the lab \| \| 462 \| We probably not get that funding \| \| 463 \| Sort of driving and supporting \| \| 464 \| The department is actually paying the rent \| \| 465 \| Having microphone, headphone and video cameras \| \| 466 \| I received money from X so now we got this EEG equipment \| \| 467 \| They trust my experience and trust my ideas \| \| 468 \| Yes, we reach one step at a time \| \| 469 \| I have come further than I have thought \| \| 470 \| I have come further than I have thought \| \| 471 \| Collaboration with that and researcher in Norway, G, K and in L \| \| 472 \| New connection and inter-disciplinary connections \| \| 473 \| Moved now with computer science \| \| 474 \| Interested in working with people \| |
| --- | --- | --- | --- | --- | --- | --- | --- | --- | --- | --- | --- | --- | --- | --- | --- | --- | --- | --- | --- | --- | --- | --- | --- | --- | --- | --- | --- | --- | --- | --- | --- | --- | --- | --- | --- | --- | --- | --- | --- | --- | --- | --- | --- | --- | --- | --- | --- | --- | --- | --- | --- | --- | --- | --- | --- | --- | --- | --- | --- | --- | --- | --- | --- | --- | --- | --- | --- | --- | --- | --- | --- | --- | --- | --- | --- | --- | --- | --- | --- | --- | --- | --- | --- | --- | --- | --- | --- | --- | --- | --- | --- | --- | --- | --- | --- | --- | --- | --- | --- | --- | --- | --- | --- | --- | --- | --- | --- | --- | --- | --- | --- | --- | --- | --- | --- | --- | --- | --- | --- | --- | --- | --- | --- | --- | --- | --- | --- | --- | --- | --- | --- | --- | --- | --- | --- | --- | --- | --- | --- | --- | --- | --- | --- | --- | --- | --- | --- | --- | --- | --- | --- | --- | --- | --- | --- | --- | --- | --- | --- | --- | --- | --- | --- | --- | --- | --- | --- | --- | --- | --- | --- | --- | --- | --- | --- | --- | --- | --- | --- | --- | --- | --- | --- | --- | --- | --- | --- | --- | --- | --- | --- | --- | --- | --- | --- | --- | --- | --- | --- | --- | --- | --- | --- | --- | --- | --- | --- | --- | --- | --- | --- | --- | --- | --- | --- | --- | --- | --- | --- | --- | --- | --- | --- | --- | --- | --- | --- | --- | --- | --- | --- | --- | --- | --- | --- | --- | --- | --- | --- | --- | --- | --- | --- | --- | --- | --- | --- | --- | --- | --- | --- | --- | --- | --- | --- | --- | --- | --- | --- | --- | --- | --- | --- | --- | --- | --- | --- | --- | --- | --- | --- | --- | --- | --- | --- | --- | --- | --- | --- | --- | --- | --- | --- | --- | --- | --- | --- | --- | --- | --- | --- | --- | --- | --- | --- | --- | --- | --- | --- | --- | --- | --- | --- | --- | --- | --- | --- | --- | --- | --- | --- | --- | --- | --- | --- | --- | --- | --- | --- | --- | --- | --- | --- | --- | --- | --- | --- | --- | --- | --- | --- | --- | --- | --- | --- | --- | --- | --- | --- | --- | --- | --- | --- | --- | --- | --- | --- | --- | --- | --- | --- | --- | --- | --- | --- | --- | --- | --- | --- | --- | --- | --- | --- | --- | --- | --- | --- | --- | --- | --- | --- | --- | --- | --- | --- | --- | --- | --- | --- | --- | --- | --- | --- | --- | --- | --- | --- | --- | --- | --- | --- | --- | --- | --- | --- | --- | --- | --- | --- | --- | --- | --- | --- | --- | --- | --- | --- | --- | --- | --- | --- | --- | --- | --- | --- | --- | --- | --- | --- | --- | --- | --- | --- | --- | --- | --- | --- | --- | --- | --- | --- | --- | --- | --- | --- | --- | --- | --- | --- | --- | --- | --- | --- | --- | --- | --- | --- | --- | --- | --- | --- | --- | --- | --- | --- | --- | --- | --- | --- | --- | --- | --- | --- | --- | --- | --- | --- | --- | --- | --- | --- | --- | --- | --- | --- | --- | --- | --- | --- | --- | --- | --- | --- | --- | --- | --- | --- | --- | --- | --- | --- | --- | --- | --- | --- | --- | --- | --- | --- | --- | --- | --- | --- | --- | --- | --- | --- | --- | --- | --- | --- | --- | --- | --- | --- | --- | --- | --- | --- | --- | --- | --- | --- | --- | --- | --- | --- | --- | --- | --- | --- | --- | --- | --- | --- | --- | --- | --- | --- | --- | --- | --- | --- | --- | --- | --- | --- | --- | --- | --- | --- | --- | --- | --- | --- | --- | --- | --- | --- | --- | --- | --- | --- | --- | --- | --- | --- | --- | --- | --- | --- | --- | --- | --- | --- | --- | --- | --- | --- | --- | --- | --- | --- | --- | --- | --- | --- | --- | --- | --- | --- | --- | --- | --- | --- | --- | --- | --- | --- | --- | --- | --- | --- | --- | --- | --- | --- | --- | --- | --- | --- | --- | --- | --- | --- | --- | --- | --- | --- | --- | --- | --- | --- | --- | --- | --- | --- | --- | --- | --- | --- | --- | --- | --- | --- | --- | --- | --- | --- | --- | --- | --- | --- | --- | --- | --- | --- | --- | --- | --- | --- | --- | --- | --- | --- | --- | --- | --- | --- | --- | --- | --- | --- | --- | --- | --- | --- | --- | --- | --- | --- | --- | --- | --- | --- | --- | --- | --- | --- | --- | --- | --- | --- | --- | --- | --- | --- | --- | --- | --- | --- | --- | --- | --- | --- | --- | --- | --- | --- | --- | --- | --- | --- | --- | --- | --- | --- | --- | --- | --- | --- | --- | --- | --- | --- | --- | --- | --- | --- | --- | --- | --- | --- | --- | --- | --- | --- | --- | --- | --- | --- | --- | --- | --- | --- | --- | --- | --- | --- | --- | --- | --- | --- | --- | --- | --- | --- | --- | --- | --- | --- | --- | --- | --- | --- | --- | --- | --- | --- | --- | --- | --- | --- | --- | --- | --- | --- | --- | --- | --- | --- | --- | --- | --- | --- | --- | --- | --- | --- | --- | --- | --- | --- | --- | --- | --- | --- | --- | --- | --- | --- | --- | --- | --- | --- | --- | --- | --- | --- | --- | --- | --- | --- | --- | --- | --- | --- | --- | --- | --- | --- | --- | --- | --- | --- | --- | --- | --- | --- | --- | --- | --- | --- | --- | --- | --- | --- | --- | --- | --- | --- | --- | --- | --- | --- | --- | --- | --- | --- | --- | --- | --- | --- | --- | --- | --- | --- | --- | --- | --- | --- | --- | --- | --- | --- | --- | --- | --- | --- | --- | --- | --- | --- | --- | --- | --- | --- | --- | --- | --- | --- | --- | --- | --- | --- | --- | --- | --- | --- | --- | --- | --- | --- | --- | --- | --- | --- | --- | --- | --- | --- | --- | --- | --- | --- | --- | --- | --- | --- | --- | --- | --- | --- | --- | --- | --- | --- | --- | --- | --- | --- | --- | --- | --- | --- | --- | --- | --- | --- | --- | --- | --- | --- | --- | --- | --- | --- | --- | --- | --- | --- | --- | --- | --- | --- | --- | --- | --- | --- | --- | --- | --- | --- | --- | --- | --- | --- | --- | --- | --- |
