# Supplementary material for: Digital Health Testbeds in Sweden: An exploratory study
Source: Digit Health. 2022 Feb 14;8:20552076221075194. doi: 10.1177/20552076221075194 (PMC8848084; doi:10.1177/20552076221075194)
Supplement: sj-docx-2-dhj-10.1177_20552076221075194 - Supplemental material for Digital Health Testbeds in Sweden: An exploratory study [file sj-docx-2-dhj-10.1177_20552076221075194.docx]

Appendix B

| **No.** | **Parent Theme** | **Theme** | **Concept** | **Testbed** |
| --- | --- | --- | --- | --- |
| 1 | **1. Primary function of the testbeds** | 1. Testbed as a context | An infrastructure | TB_6,7,15_ |
| 2 |  |  | An (physical/virtual) environment | TB_9,10,11,14_ |
| 3 |  | 2. A collaborative meeting place | A collaboration arena | TB_2_ |
| 4 |  |  | A big digital playground | TB_2_ |
| 5 |  |  | A meeting place | TB_2,6,8_ |
| 6 |  |  | An arena | TB_2,13_ |
| 7 |  |  | Collaborative environment | TB_6_ |
| 8 |  |  | A venue | TB_8_ |
| 9 |  |  | A node with different functions | TB_13_ |
| 10 |  | 3. A technological testing place | Test infrastructure | TB_2_ |
| 11 |  |  | Test environment | TB_6,7_ |
| 12 |  |  | A testing company | TB_7_ |
| 13 |  |  | A living lab | TB_11_ |
| 14 |  |  | Design-driven lab | TB_14_ |
| 15 |  |  | An innovation hub | TB_14_ |
| 16 |  |  | An application hub | TB_14_ |
| 17 |  |  | A lab | TB_15_ |
| 18 | **2. Stakeholder as an organization** | 4. Academic institute | University | TB_2,4,5,6,7,8,9,10,11,12,14,15_ |
| 19 |  |  | Academia | TB_2,8,10_ |
| 20 |  | 5. Research institute/ industry | Research institute | TB_1,2,3,4,12,13,14,15_ |
| 21 |  |  | Research industry | TB_6,7_ |
| 22 |  |  | Science park/ city | TB_4,9_ |
| 23 |  | 6. Business organization | Business organization/ sector | TB_2,4,8,9,11,12_ |
| 24 |  |  | Company | TB_1,5,6,14_ |
| 25 |  |  | Business hub | TB_13_ |
| 26 |  | 7. Government organization | Government organization/ sector/ agency | TB_3,7,13,14_ |
| 27 |  |  | Municipality unit | TB_6,10_ |
| 28 |  |  | County council | TB_8,10_ |
| 29 |  | 8. Healthcare organization | Elderly care | TB_3,6,8,9,10_ |
| 30 |  |  | Healthcare (clinics)/ health center | TB_6,9,11_ |
| 31 |  | 9. Public organization | Public organization/ sector | TB_2,4,11,12_ |
| 32 |  |  | Civil organization | TB_12_ |
| 33 |  |  | Pensioners' organization | TB_8_ |
| 34 | **3. Stakeholder as an individual/ group** | 10. Stakeholder (as an individual) | Industry expert | TB_6_ |
| 35 |  |  | Technical analyst | TB_6_ |
| 36 |  |  | Market analyst | TB_6_ |
| 37 |  |  | Healthcare system developer | TB_7_ |
| 38 |  |  | Healthcare professional | TB_8_ |
| 39 |  |  | Innovator | TB_8_ |
| 40 |  |  | Researcher | TB_9_ |
| 41 |  |  | Entrepreneur | TB_12_ |
| 42 |  |  | Service designer | TB_14_ |
| 43 |  |  | Psychologist | TB_15_ |
| 44 |  |  | Scientist | TB_15_ |
| 45 |  | 11. Stakeholder (as a group of individuals) | Reference group | TB_13_ |
| 46 |  |  | Research (informal) group | TB_14_ |
| 47 | **4. Tool used to design or implement the testbed** | 12. Theory/ model | Theory of multiple view geometry | TB_1_ |
| 48 |  |  | Value proposition theory | TB_4_ |
| 49 |  |  | Google sprint design | TB_5_ |
| 50 |  |  | Service design | TB_5,8,12,14_ |
| 51 |  |  | Context approach | TB_7_ |
| 52 |  |  | Double Diamond design | TB_10,12_ |
| 53 |  |  | Business (Canvas) model | TB_10,13_ |
| 54 |  |  | Need analysis | TB_10,11,13_ |
| 55 |  |  | Kano model for developing solution | TB_10_ |
| 56 |  |  | Quadruple helix model | TB_12_ |
| 57 |  |  | Experience-based design | TB_14,15_ |
| 58 |  | 13. Strategy/ principle | Open collaboration | TB_2_ |
| 59 |  |  | Plug-and-play playground | TB_2_ |
| 60 |  |  | Not own anything while offering the latest technology | TB_2_ |
| 61 |  |  | Re-creating the testbeds for each purpose | TB_3_ |
| 62 |  |  | Adaptation to the entrepreneurial line of thoughts | TB_3_ |
| 63 |  |  | Designing the solution with the elderly in mind | TB_3_ |
| 64 |  |  | Agenda 2030 | TB_4_ |
| 65 |  |  | Agile development teams | TB_5_ |
| 66 |  |  | Using employees' experience to set up solutions | TB_7_ |
| 67 |  |  | Involving all partners in the design of the testbed | TB_8_ |
| 68 |  |  | Creating customized meetings for testing with experts | TB_8_ |
| 69 |  |  | User involvement for ensuring usability | TB_12,14_ |
| 70 |  |  | Creating value in healthcare & people's life | TB_14_ |
| 71 |  | 14. Method/ methodology | Lean start-up | TB_5_ |
| 72 |  |  | Market survey | TB_7_ |
| 73 |  |  | External monitoring | TB_9_ |
| 74 |  |  | Clinical Trial | TB_11_ |
| 75 |  |  | Pilot study | TB_11,15_ |
| 76 |  | 15. Technology implementation | Light Field display and software | TB_1_ |
| 77 |  |  | Internet of things | TB_2,5_ |
| 78 |  |  | 5G wireless communication | TB_6_ |
| 79 |  |  | EEG & Event-related Potential (ERP) | TB_15_ |
| 80 | **5. The reason behind using such tools** | 16. Based on the needs of the users/owners | To ensure the real needs of the users | TB_10,12,14_ |
| 81 |  |  | To ensure the real needs of the owners | TB_13_ |
| 82 |  | 17. Based on the previous experience | Proven as successful methods previously ( by others) | TB_5_ |
| 83 |  |  | Proven as successful by previous experience | TB_7,15_ |
| 84 |  | 18. Based on the project goal | To form a digital/ physical environment | TB_2,9_ |
| 85 |  |  | To fit well with the project focus | TB_3_ |
| 86 |  | 19. Based on the business goal | To speed up commercialization | TB_4_ |
| 87 |  |  | To find the right customers | TB_13_ |
| 88 |  | 20. Based on the evaluation | To ensure usability by evaluation (during the project) | TB_8_ |
| 89 |  |  | To ensure usability by evaluation (before the project) | TB_11_ |
| 90 | **6. Purpose of the testbeds** | 21. Improve the quality of care delivery or service | Quality | TB_1,4,6,7,10,12,14_ |
| 91 |  |  | Safety (patient/ community) | TB_1,2,14_ |
| 92 |  |  | Efficiency | TB_1,3,12_ |
| 93 |  |  | Patient-centeredness | TB_7,8,11_ |
| 94 |  |  | Effectiveness | TB_3,9_ |
| 95 |  |  | Availability | TB_2_ |
| 96 |  |  | Equity | TB_12_ |
| 97 |  | 22. Develop, test or create demand for eHealth solution, product or service | Clinical application | TB_1_ |
| 98 |  |  | New (sustainable) product/ solution | TB_2,4,9,11,12,13_ |
| 99 |  |  | Digital healthcare service | TB_11,14_ |
| 100 |  |  | Technical innovation | TB_7,10_ |
| 101 |  |  | Create demand for eHealth solutions or service | TB_10,13,14_ |
| 102 |  | 23. Build collaborative environment | Sustainable collaboration environment | TB_2,6_ |
| 103 |  |  | Regional (inter) collaboration | TB_5,14_ |
| 104 |  |  | Innovator friendly climate | TB_8_ |
| 105 |  |  | A consortium | TB_13_ |
| 106 |  |  | New research collaboration | TB_15_ |
| 107 |  | 24. Develop and support business growth | Growth for small and medium-sized companies | TB_3,6,11_ |
| 108 |  |  | Regional business | TB_9_ |
| 109 |  |  | Private business | TB_12_ |
| 110 |  | 25. Develop research and education | Knowledge through research and education | TB_4_ |
| 111 |  |  | Research environment (area) | TB_5,15_ |
| 112 |  |  | Opportunities for research and education | TB_15_ |
| 113 | **7. Facilitation factor** | 26. Facilitation of research and education oriented activities | Research | TB_1,11,12,14_ |
| 114 |  |  | Training | TB_13,14,15_ |
| 115 |  |  | Education | TB_12,14_ |
| 116 |  |  | Course | TB_13,15_ |
| 117 |  |  | Workshop | TB_14,15_ |
| 118 |  |  | Competence | TB_14_ |
| 119 |  | 27. Facilitation of collaboration, communication, connection | National/ international/ transnational collaboration | TB_2,4,11,14_ |
| 120 |  |  | Collaborative environment | TB_2,3,13_ |
| 121 |  |  | Connection | TB_2,8,11_ |
| 122 |  |  | Networking of (Ph.D. students) | TB_13,14,15_ |
| 123 |  | 28. Facilitation of eHealth service (testing and evaluation) | Testing of digital product/solution | TB_1,2,3,6,7,9,10,11,12,13_ |
| 124 |  |  | Evaluation of need for a digital solution | TB_8,9,10,11,13,14_ |
| 125 |  |  | Pre-testing of digital product/solution | TB_3,7_ |
| 126 |  | 29. Facilitation of management operation | Governance by the existing line of organization | TB_3,14_ |
| 127 |  |  | Management by the appointed department | TB_5_ |
| 128 | **8. Challenging factor** | 30. Time and resource constraints | Time and resource consuming process | TB_3_ |
| 129 |  |  | Lack of people’s (healthcare professionals) time | TB_5,11_ |
| 130 |  |  | Wasting excessive time on the concept/model/definition | TB_9,10,13,14_ |
| 131 |  |  | Wasting non-productive effort on concept/model/definition | TB_9,10,13,14_ |
| 132 |  | 31. Uncertainty | Rules (to apply) | TB_2_ |
| 133 |  |  | Focus (industrial) | TB_4_ |
| 134 |  |  | Concept | TB_9_ |
| 135 |  |  | Goal (vague) | TB_11_ |
| 136 |  |  | Business model | TB_10,13_ |
| 137 |  |  | Definition | TB_13,14_ |
| 138 |  |  | Future of healthcare | TB_14_ |
| 139 |  | 32. Communication related | Lack of involvement of the stakeholders | TB_5,9,10,11_ |
| 140 |  |  | Lack of communication | TB_5_ |
| 141 |  |  | Distance between the stakeholders | TB_7,14_ |
| 142 |  | 33. Policy and guideline related | Rules | TB_2_ |
| 143 |  |  | Legal guidance (aspects) | TB_8,10,12_ |
| 144 |  |  | Collection of agreement (relevant) documents | TB_8,12_ |
| 145 |  |  | Developing a payment model (set up a fee) | TB_8,10_ |
| 146 |  | 34. Technical (implementation) challenges | Controls over testbed details | TB_1_ |
| 147 |  |  | Technology itself that prevents finding solutions | TB_7_ |
| 148 |  |  | Long time to read (volume of) a technical description | TB_7_ |
| 149 |  |  | Excessive cost of implementation | TB_7_ |
| 150 |  |  | Demographic challenge | TB_10_ |
| 151 |  |  | Research technical issues & inadequate research equipment | TB_15_ |
| 152 |  | 35. Teamwork related | Commitment from (top) management (issue) | TB_3,14_ |
| 153 |  |  | Trust between stakeholders | TB_5_ |
| 154 |  |  | Frustration of employee during implementation | TB_7_ |
| 155 |  |  | Heavy workload & stress | TB_14_ |
| 156 |  | 36. Environment related | Challenges of healthcare while performing research | TB_10,12,14_ |
| 157 |  |  | Lack of access to healthcare site for research | TB_11,12_ |
| 158 |  | 37. Productivity related | Very few (not many) tests were conducted | TB_2,11_ |
| 159 |  |  | Difficulties in (healthcare) product development | TB_11_ |
| 160 |  |  | Lack of clients (buyers) | TB_5_ |
| 161 |  | 38. Organizational (and sustainability) challenge | Matching organizational needs with entrepreneurial ideas | TB_3_ |
| 162 |  |  | Difficult to be known as an organization | TB_9_ |
| 163 |  |  | Sustainability of the testbed project | TB_4_ |
| 164 |  |  | Sustainability of the testbed environment | TB_11_ |
| 165 |  | 39. Workforce related | Retain skilful staff and experts | TB_11_ |
| 166 |  |  | Shortage of staff | TB_14_ |
| 167 | **9. Ameliorating factor** | 40. Additional funding | Received (partial) additional funding from the government | TB_8,11_ |
| 168 |  |  | Received additional funding from an innovation agency | TB_9,10,14_ |
| 169 |  |  | Received additional funding from a private funding agency | TB_15_ |
| 170 |  | 41. Learning and insight | Shared lessons learned | TB_2_ |
| 171 |  |  | Created learning opportunities | TB_3_ |
| 172 |  |  | Acquired learnings and insights (from the results) | TB_10,12,14_ |
| 173 |  | 42. Technical implementation | Took full control testbed details (researchers) | TB_1_ |
| 174 |  |  | Provided stand-alone test scripts for better implementation | TB_7_ |
| 175 |  |  | Bought extra research equipment | TB_14_ |
| 176 |  |  | Implemented commercialisation | TB_4_ |
| 177 |  | 43. Teamwork | Empowered colleagues to gain new experiences & tools | TB_5_ |
| 178 |  |  | Focused on limited cases for having concrete idea | TB_2_ |
| 179 |  |  | Research team was granted permit to work at the hospital | TB_12_ |
| 180 |  | 44. Policy and guideline | Produced legal guidance | TB_8_ |
| 181 |  |  | Appointed an evaluation researcher for guidance | TB_8_ |
| 182 |  | 45. Collaboration | Appointed a coordinator for international stakeholders | TB_11_ |
| 183 |  |  | Participated in other funded projects | TB_11_ |
| 184 |  | 46. Organisation (and sustainability) related | Internal re-organisation | TB_2_ |
| 185 |  |  | Reorganised & rebuilt a stronger facilitation process | TB_14_ |
| 186 |  |  | Made the testbeds last with sustainable value propositions | TB_4_ |
| 187 |  |  | Reached to a sustainable business model | TB_13_ |
| 188 | **10. Outcome of the testbed projects** | 47. Project goals were achieved *mostly* | Achieved most of the project goals | TB_10,11_ |
| 189 |  |  | Achieved number of deliverables/assignments/innovations | TB_1,3,5,7,8,9,12,13_ |
| 190 |  | 48. Project goals were achieved *completely* | Became a collaboration arena | TB_2_ |
| 191 |  |  | Became a regular line organisation | TB_9_ |
| 192 |  |  | A big success with national/international impact | TB_14_ |
| 193 |  |  | Achieved more results than anticipated | TB_15_ |
| 194 |  | 49. Project is still ongoing | Project is still ongoing | TB_4,6,12_ |
